# Does electrical stimulation in the lower urinary tract increase urine production? A randomised comparative proof-of-concept study in healthy volunteers

**DOI:** 10.1371/journal.pone.0217503

**Published:** 2019-05-24

**Authors:** Stéphanie van der Lely, Martina D. Liechti, Werner L. Popp, Melanie R. Schmidhalter, Thomas M. Kessler, Ulrich Mehnert

**Affiliations:** 1 Department of Neuro-Urology, Balgrist University Hospital, University of Zürich, Zürich, Switzerland; 2 Rehabilitation Engineering Lab, ETH Zürich, Zürich, Switzerland; 3 Spinal Cord Injury Center, Balgrist University Hospital, University of Zürich, Zürich, Switzerland; University Medical Center Utrecht, NETHERLANDS

## Abstract

**Trial design:**

During electrical stimulation in the lower urinary tract for the purpose of current perception threshold and sensory evoked potential recording, we observed that bladder volume increased rapidly. The aim of this prospective randomised comparative proof-of-concept study was to quantify urine production per time during stimulation of the lower urinary tract using different stimulation frequencies.

**Methods:**

Ninety healthy subjects (18 to 36 years old) were included. Forty females and 50 males were randomly assigned to one of the following study groups: dome, trigone or proximal, membranous (males only) or distal urethra. Starting from 60mL prefilling, stimulation was performed at two separate visits with a 14 French custom-made catheter using randomly applied frequencies of 0.5Hz, 1.1Hz, 1.6Hz (each with 500 stimuli). After each stimulation cycle per frequency, urine production was assessed. Main outcome measures represented urine production during stimulation, daily life and their ratio.

**Results:**

Lower urinary tract electrical stimulation increased urine production per time compared to bladder diary baseline values. Linear mixed model showed that frequency (p<0.001), stimulation order (p = 0.003), intensity (p = 0.042), and gender (p = 0.047) had a significant influence on urine production. Location, visit and age had no significant influence.

**Conclusions:**

Urine production is increased during electrical stimulation with a bigger impact of higher frequencies. This might be relevant for methodological aspects in the assessment of lower urinary tract afferent function and for patients with impaired renal urine output. Inhibition of renal sympathetic nerve activity by vagal afferents may be the underlying mechanism.

## Introduction

Current perception threshold (CPT) and sensory evoked potential (SEP) recording are established techniques in neurophysiology to test human afferent nerve function and integrity, respectively. This also seems to be a promising approach for advanced sensory assessment of the lower urinary tract (LUT), which our group has investigated in healthy women and men with normal bladder function [1, 2]. Interestingly, during such neurophysiological studies using electrical LUT stimulation, we frequently observed that bladder volume seemed to increase rapidly over a short period of time. Apart from an online presentation that mentioned a similar observation during the assessment of bladder electrical stimulation on urine production in patients with acute decompensated heart failure [3], the literature on this topic is scarce and there is a lack of knowledge regarding functional interrelation of LUT stimulation and renal urine production. Thus, in this proof-of-concept study we aimed to quantify and validate our observations in terms of different stimulation frequencies, intensities and LUT locations. This is relevant from a physiological point of view since there is not yet a clear concept on the relationship between LUT electrical stimulation and urine production per time (UPT), which may be of clinical interest for diuretic treatment in cardio-vascular pathologies. Furthermore, it is important for measurements such as LUT CPT [4, 5] and SEP recording [1, 2] because rapidly changing bladder volumes may affect desire to void sensation altering susceptibility for electrical current as well as measurement accuracy due to electrode dislocation from the expanding bladder wall [6].

Based on our observations, we hypothesized that LUT stimulation would increase UPT compared to baseline values. The increase in UPT was expected to be larger when stimulating with higher frequency/absolute stimulation intensity (STIMINT), compared to lower frequency/STIMINT due to enhanced energy input per time with higher frequencies/STIMINT. Regarding location specific innervation, UPT was expected to be higher during stimulation of trigone due to the higher density of neuronal innervation in this area [7, 8].

## Materials and methods

This prospective parallel-group study was approved by the local ethics committee (Kantonale Ethikkommission Zürich), registered at clinicaltrials.gov (Identifier: NCT02272309), and performed in accordance with the Declaration of Helsinki. Data were collected and managed using REDCap electronic data capture tools [9]. All subjects provided written informed consent prior to inclusion.

### Study design

**Fig 1:**
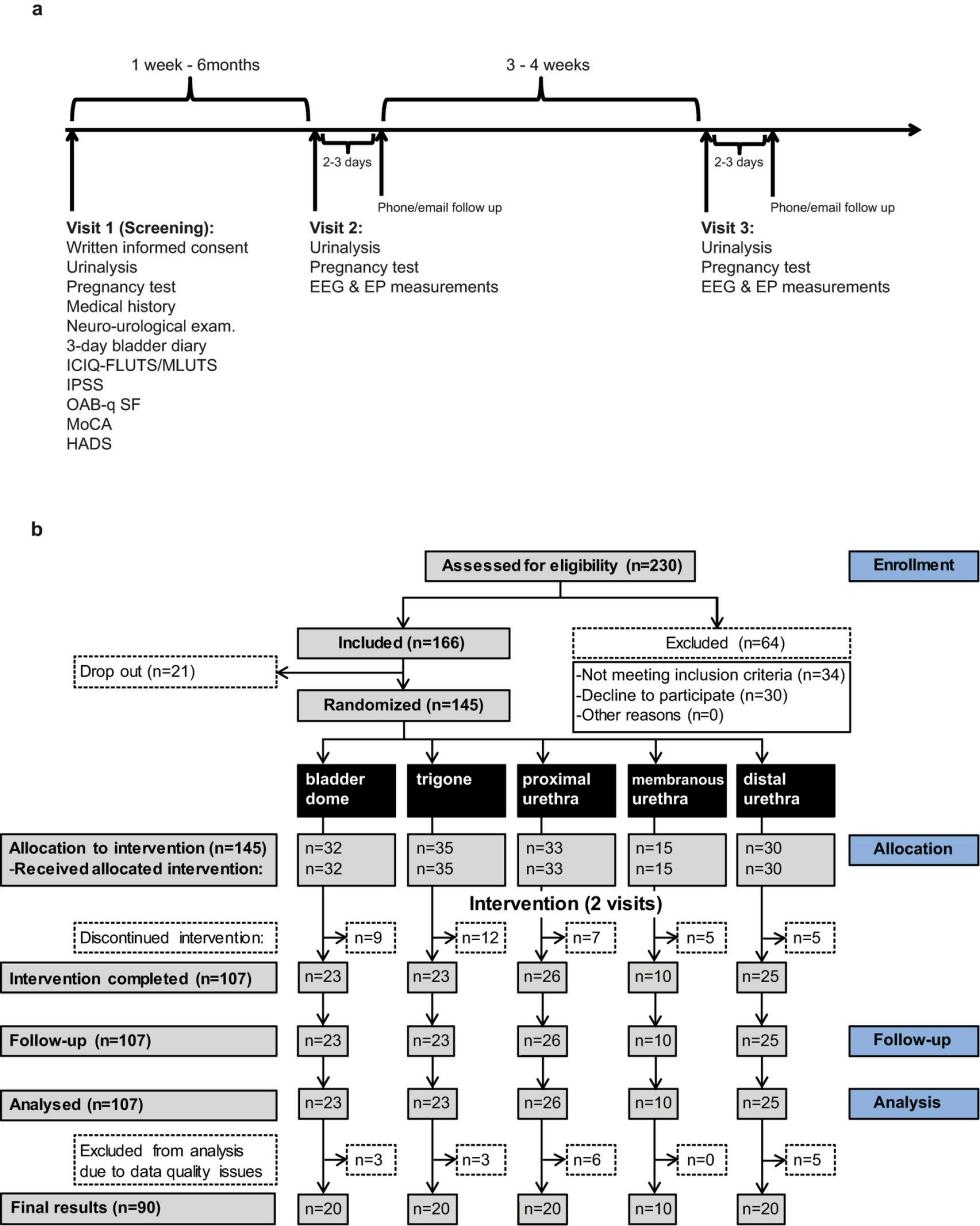
**Study design and time schedule (a) and Consort diagram for flow of participants through the study (b).** Reasons for discontinued intervention were: no participation in visit 2 (number of subjects: n = 18), catheter could not be placed (n = 14), uncomfortable feeling caused by catheter/stimulation (n = 5), poor health condition (n = 1). HADS: Hospital Anxiety and Depression Scale; ICIQ-FLUTS: International Consultation on Incontinence Modular Questionnaire Female lower urinary tract symptoms; ICIQ-MLUTS: International Consultation on Incontinence Modular Questionnaire Male lower urinary tract symptoms; IPSS: International Prostate Symptom Score; MoCA: Montreal Cognitive Assessment; OAB-q SF: The Overactive Bladder Questionnaire short-form.

**Fig 2:**
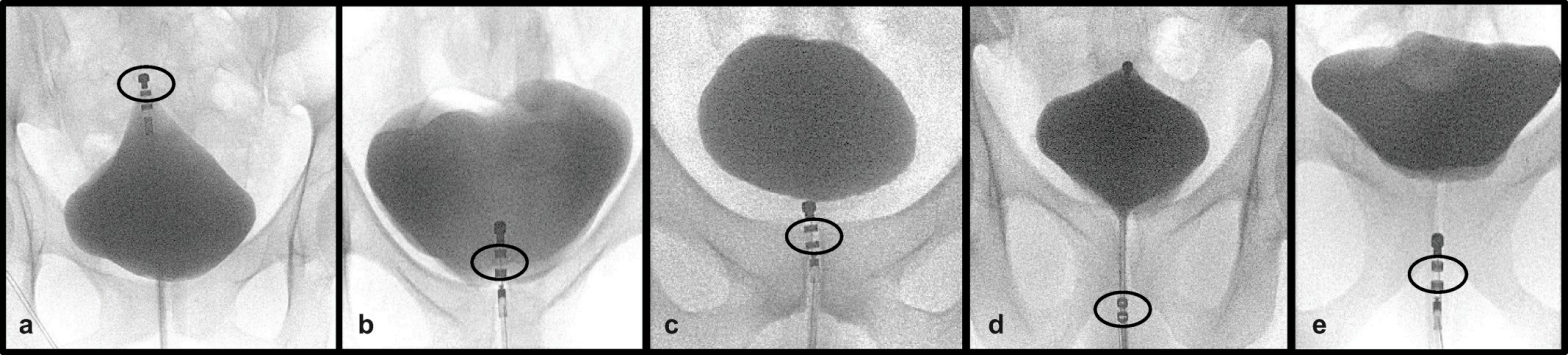
Fluoroscopic images for catheter positioning at different stimulation locations. Examples of fluoroscopic images taken after catheter positioning at the five specific stimulation locations: Bladder dome (BD, 2a), trigone (TG, 2b), proximal urethra (pUR, 2c), membranous urethra (mUR, 2d), distal urethra (dUR, 2e). Bladder volume was 60mL of contrast medium. Radiographs 2a, 2b, and 2e show positioning of the catheter for stimulation in female lower urinary tract (LUT), while 2c and 2d represent images of the catheter in male LUT. The stimulating electrodes are encircled in black.

This proof-of-concept study was embedded in the frame of a LUTSEP study (S1 Study protocol) [10]. The corresponding power analysis outlined in our protocol paper [10] revealed a total inclusion number of 90 subjects. Forty females and fifty males were scheduled for two separate (interval of 29.0±8.5days) but identical visits and randomly assigned to one of the following LUT stimulation groups (1:1:1:1:1): bladder dome (BD), trigone (TG), proximal urethra (pUR), membranous urethra (mUR, additional location in males considering gender-specific anatomical characteristics), and distal urethra (dUR) (Figs 1 and 2).

This proof-of-concept randomised clinical trial aimed to determine feasibility of volumetric assessments (i.e. UPT) during LUTSEP recordings in order to inform a planned study on *The Effect of Lower Urinary Tract Electrical Stimulation on Renal Urine Production (Diuresis)* (see corresponding trial registration, NCT03256656).

### Subjects

Participants recruited via announcements at the University of Zürich, in local print and online media, were invited for screening assessments to the Neuro-Urology, Spinal Cord Injury Centre at Balgrist University Hospital between October 2015 and June 2017. Inclusion criteria were age between 18 and 40 years, good mental and physical health. Exclusion criteria were lower urinary tract symptoms (LUTS) [11], urological or neurological pathology, pregnancy, current or recurrent urinary tract infection (UTI), hematuria, previous surgery for urological or neurological reasons, and regular intake of any kind of prescribed or non-prescribed medication (except contraceptives). This was assessed on the basis of a complete medical history interview, vital signs, physical and neurological examinations (including examination of urogenital sensation, bulbocavernosus reflex, anal reflex, anal sphincter tone, and anal squeeze response), free uroflowmetry, post-void residual, Montreal-Cognitive-Assessment (MoCA), Hospital Anxiety and Depression Scale (HADS), International Prostate Symptom Score (IPSS) and a 3-day bladder diary (BLD) [10] using predefined cut-offs (Table 1).

**Table 1:** Baseline characteristics (n = 90, 40 females).

Baseline characteristics	Women (n = 40)	Men (n = 50)	All (n = 90)	p Value—gender	p Value—locations
***Age [years]*** ^b^	23.5 (18.3–35.8)	23.6 (18.3–34.1)	23.6 (18.3–35.8)	0.581	0.553
***Height [m]*** ^b^	1.7 (1.6–1.9)	1.8 (1.6–2.0)	1.7 (1.6–2.0)	<0.001*	0.163
***Weight [kg]*** ^b^	61 (48–85)	74.5 (57–126)	67.5 (48–126)	<0.001*	0.212
***3-day bladder diary***					
Micturition frequency per 24 hours^a^	6.5±1.7	5.2±1.9	5.8±1.9	0.001*	0.909
Micturition volume per micturition [mL] ^b^	293 (162–718)	339 (209–1057)	325 (162–1057)	0.112	0.534
Fluid intake per 24 hours [mL] ^b^	2140 (1050–5717)	2115 (783–7953)	2117 (783–7953)	0.987	0.484
***Questionnaires***					
ICIQ-FLUTS/MLUTS^+^					
Filling symptoms ^b^	1 (0–5)	.	.		0.867
Voiding symptoms ^b^	0 (0–3)	1 (0–6)	.		0.178/0.825
Incontinence symptoms ^b^	0 (0–2)	0.5 (0–4)	.		0.539/0.694
IPSS ^b^	.	1 (0–6)	.		0.611
OAB-q SF					
Symptoms ^b^	6 (6–11)	6 (6–16)	6 (6–16)	0.013*	0.340
QoL ^b^	13 (13–17)	13 (13–18)	13 (13–18)	0.188	0.570
HADS					
Anxiety ^b^	3.5 (0–7)	3 (0–7)	3 (0–7)	0.086	0.396
Depression ^b^	1 (0–6)	1 (0–6)	1 (0–6)	0.949	0.558
MoCA ^b^	28.5 (26–31)	29 (26–30)	29 (26–31)	0.802	0.655
***Neuro-Urological examination***					
Urogenital sensation (n intact/impaired)	40/0	50/0	90/0		
Bulbocavernosus reflex (n intact/impaired)	40/0	49/1	89/1		
Anal reflex (n intact/impaired)	40/0	50/0	90/0		
Anal sphincter tone (n intact/impaired)	40/0	50/0	90/0		
Anal squeeze response (n intact/impaired)	40/0	50/0	90/0		
***Free uroflowmetry***					
Voided volume [mL] ^b^	448 (161–1243)	393 (95–1195)	421 (95–1243)	0.600	0.394
Maximum flow rate [mL/s] ^b^	39.4 (12.4–79.4)	30.6 (11.1–77.4)	34.0 (11.1–79.4)	0.002*	0.227
Post void residual [mL] ^b^	1.5 (0–64.5)	3.2 (0–117)	2.7 (0–117)	0.190	0.821

Data are represented as

(^a^) mean±standard deviation (SD) or

(^b^) median (range: minimum-maximum) or number of subjects (n) if appropriate.

All subjects fulfilled predefined cut-offs for study inclusion: MoCA score ≥26, HADS ≤7 each, IPSS ≤7, BLD: $\frac{24h\;urinary\;frequency}{drinking\;volume\;[mL]}\leq 0.0045$ with a maximum of 1x nocturia, mean volume per void >150mL and absence of urinary incontinence or urgency.

Asterisk (*) indicates statistical significance p<0.05.

^+^ due to different scoring systems, female and male subjects have not been compared. Significances were comparable when excluding the location mUR. ICIQ = International Consultation on Incontinence Modular Questionnaire, FLUTS = Female lower urinary tract symptoms, MLUTS = Male lower urinary tract symptoms, IPSS = International Prostate Symptom Score, OAB-q SF = The Overactive Bladder Questionnaire short-form, QoL = Quality of life, HADS = Hospital Anxiety and Depression Scale, MoCA = Montreal Cognitive Assessment.

Descriptive statistics stratified for stimulation location and gender are reported in S1A–S1E Table.

The BLDs were completed during three independent days, recording the time points and volumes (mL) of drinking and micturition, as well as the number of incontinence episodes, pad usage, and pain levels associated with urine storage and/or micturition (0 to 10). Additionally, standardized urological questionnaires (International Consultation on Incontinence Modular Questionnaire modules (ICIQ-FLUTS, ICIQ-MLUTS) and Overactive Bladder Questionnaire short-form (Swiss German OAB)) were completed [10]. All questionnaires and the bladder diary were independently completed by the participants.

### Procedures

All subjects were instructed to adhere to their usual liquid consumption according to their bladder diary, avoiding, however, consumption of caffeine and cigarettes three hours and alcohol one day prior to the measurement. Prior to experimental procedures, pregnancy test and urine dip stick (Combur-Test) analysis were performed. The daytime of investigation was held constant across visits between 0 to 3h. The room temperature during measurements was kept constant at 23±1°C.

Constant current stimulation was generated using a neurophysiological stimulator (Dantec Keypoint Focus, Neurolite AG, Belp, Switzerland) and applied via a transurethrally placed custom-made stimulation catheter (14 French, Unisensor AG, Attikon, Switzerland) [10]. After catheter insertion, the bladder was emptied and refilled with 60mL of contrast medium (Ultravist 150, Bayer AG, Switzerland). The radiopaque electrodes and markers on the catheter were used to ensure correct positioning under fluoroscopic guidance (Fig 2) [1].

CPTs were identified according to the methods of limits [12]. After pain threshold assessment, STIMINT was individually decreased aiming to have a tolerable but non-painful sensation. A total number of 500 square wave stimuli were applied in 3 cycles, each with a different frequency, i.e. 0.5Hz, 1.1Hz, and 1.6Hz (each 1ms pulse width). Following a repeated-measures, randomised controlled factorial design, the frequencies were pseudorandomly applied using a computer-generated randomization list stratified on gender. Sequence generation and randomisation was performed by the research team, who were not formally blinded to group allocation.

After each stimulation cycle, the bladder was emptied and volumes were recorded. Additionally, the time of bladder emptying/filling and the start/end time of electrical stimulation was recorded. Wellbeing and adverse events of each subject were assessed immediately and followed up in a telephone interview 2–3 days after each visit. Follow-up was completed in August 2017.

### Data analysis

Urine dip stick was analysed regarding UTI and specific urine weight. The BLD values were evaluated calculating the average of each day following by averaging over the three days. In addition to the 24-hour measurements, daytime BLD values (DT1: wake-up time till bedtime, DT2: wake-up time till bedtime minus the first morning urine volume) were calculated to avoid underestimation of the baseline UPT. The mean micturition volume was converted into mL/min and used as baseline reference for the natural urine output of the subjects.

**Fig 3:**
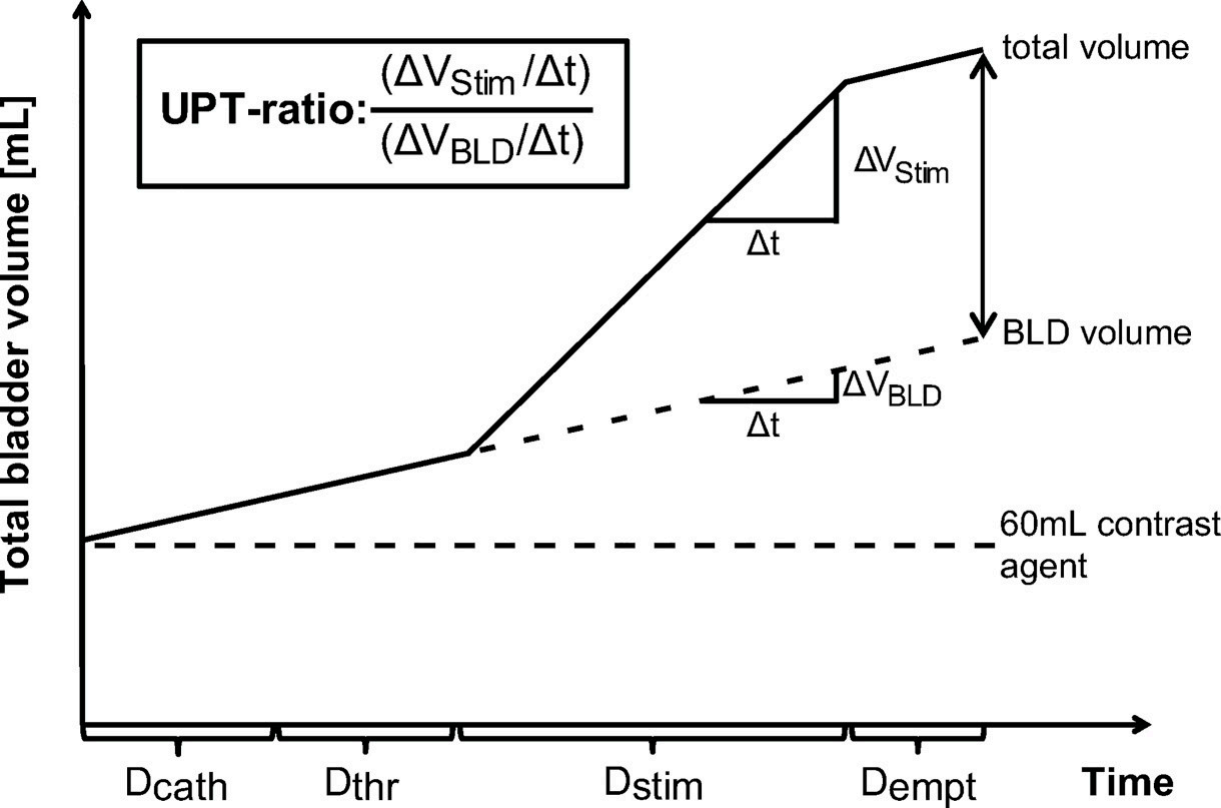
Calculation of outcome measure “Urine production per time (UPT)-ratio”. During each section (D_cath_, D_thr_, D_stim_, D_empt_) of the stimulation cycle we assumed baseline UPT based on the bladder diary (BLD) measurements (UPT_BLD_). In our model we further assumed increased UPT during electrical stimulation (UPT_Stim_). The outcome measure “UPT-ratio” was calculated by dividing UPT_Stim_ *(ΔV*_*Stim*_*/Δt)* by UPT_BLD_ *(ΔV*_*BLD*_*/Δt)*. In summary, the outcome measure describes to which factor the UPT was higher during D_stim_ compared to the baseline value from the BLD. D_cath_ = time [s] used for catheter positioning at specific stimulation location; D_thr_ = time [s] used for current perception threshold (CPT) / pain threshold assessment and definition of absolute stimulation intensity (STIMINT); D_stim_ = time [s] used for electrical stimulation (500 stimuli); D_empt_ = time [s] used for bladder emptying. ΔV = volumetric changes, Δt = time difference.

During the experimental procedures, produced volume represents the emptied volume minus the starting volume of 60mL. UPT was analysed to adjust for the variable durations (D) of the experimental procedure related to the stimulation frequencies and the individual examination sections (Dcath = catheter positioning at specific stimulation location, Dthr = perception and pain threshold assessment, Dstim = stimulation, Dempt = bladder emptying) of each stimulation cycle (Fig 3). The course of a stimulation cycle and the calculation of our main outcome measure “UPT-ratio” is illustrated in Fig 3.

### Statistical analysis

Data processing and statistical analyses were performed using RStudio (Version 1.0.136, Boston, MA, USA) and MATLAB R2017a (The MathWorks, Tatick, MA, USA). Data were examined by exploratory data analysis methods and described providing mean and standard deviation or median and range (minimum-maximum) according to the data distribution (normal vs non-normal) tested using Shapiro-Wilk test, histograms and qq-plots.

Unpaired Welch’s t-tests or Mann-Whitney-U tests and one-way ANOVA or Kruskal-Wallis test were performed to check for gender and location differences, respectively. Post-hoc comparisons (unpaired Welch’s t-tests or Mann-Whitney-U tests, significance level p<0.05) stratified for location were only reported for significant overall gender effects (p<0.05). Wilcoxon Signed-Rank tests were used to compare UPT during experimental conditions to baseline. UPT-ratio was analysed using linear mixed-effect models (LMM). As fixed effects, stimulation frequency [Hz], STIMINT [mA], stimulation location (TG versus BD, pUR, mUR, and dUR), stimulation order (1^st^ versus 2^nd^, 3^rd^ stimulation), age [years], gender (male, female), and visit (1^st^, 2^nd^ visit) were used. Additionally, the intercepts for the subjects were added as random effects. Frequency was included as continuous predictor, even if only three frequencies were tested. In order to determine the significance of the fixed effects, a simulated likelihood ratio test (LRT) with n = 10’000 replications was used where the model including the specific fixed effect was compared against the model without the specific fixed effect. For all statistical analyses, a significance level of p<0.05 was used. As supplementary analyses, LMMs were performed with adaptations to our main model: 1) without mild outliers of UPT-ratio. A mild outlier was defined as a point beyond the inner fence (quartile1-1.5*interquartile range; quartile3+1.5*interquartile range); 2) location mUR was removed (only measured in males); 3) produced volume [mL] as outcome measure; 4) including all daytime BLD values (DT1) when calculating UPT-ratio; 5) including daytime BLD values minus the first morning urine volume (DT2) when calculating UPT-ratio.

## Results

Ninety subjects (40 females, 50 males) with a median age of 23.6 years (range: 18.3–35.8 years) were included for the analysis. Baseline characteristics are shown in Table 1 and S1A–S1E Table. One subject was excluded from a few statistical analyses due to a missing urine volume value after one stimulation cycle. Subjects reported mild, temporary, and self-limited (1–5 days) dysuria after 109 out of 180 measurements (62 out of 90 subjects) and mild, temporary, and self-limited (1–3 days) haematuria after 9 out of 180 measurements (9 subjects out of 90). Otherwise, all subjects tolerated the procedures well and no symptomatic UTI was reported.

### Duration of stimulation cycle sections

For the different sections of each stimulation cycle, the following median durations were observed (Fig 3, S1 Fig): D_cath_ = 134s (10-1073s), D_thr_ = 103s (13-1108s), D_empt_ = 104s (20-1768s). D_stim_ is systematically dependent on the frequency, resulting in a stimulation time of 16.7min, 7.6min, and 5.2min for 0.5Hz, 1.1Hz, and 1.6Hz respectively.

### Urine production

**Fig 4:**
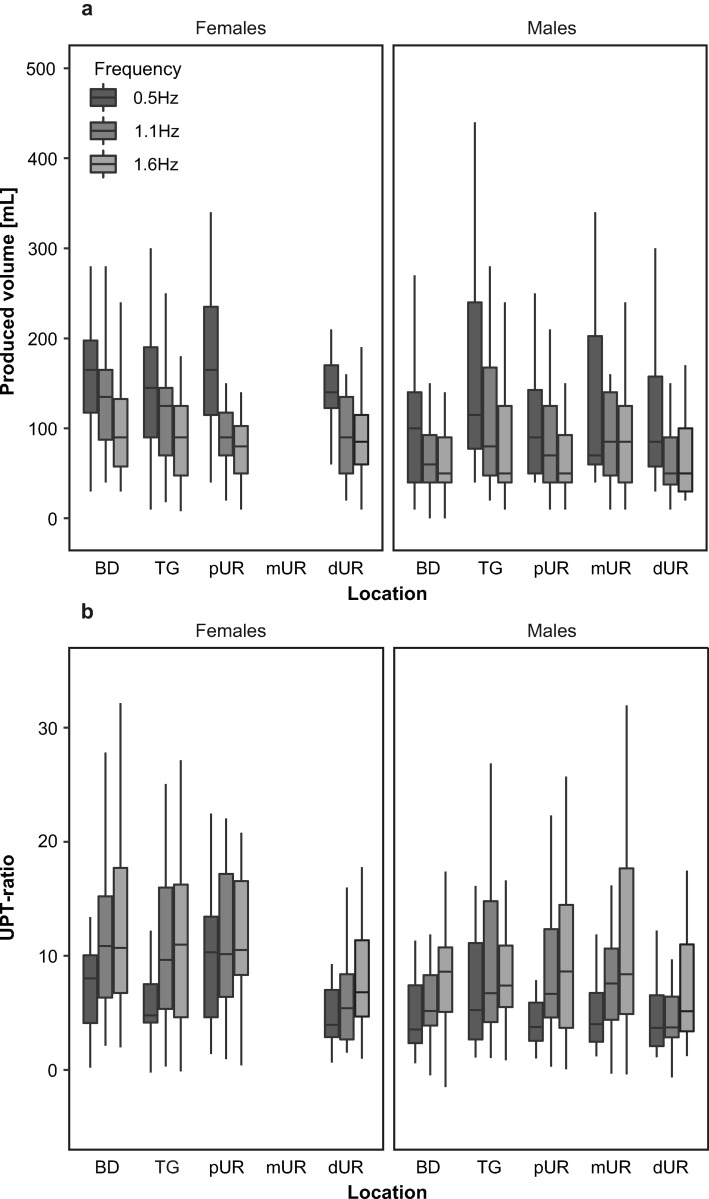
Box plots of median, 25th and 75th percentile and whiskers of urine production. Produced volume [mL] (4a) and urine production per time (UPT)-ratio (4b) are shown for the three stimulation frequencies and five stimulation sites (BD: bladder dome; TG: trigone; pUR: proximal urethra; mUR: membranous urethra; dUR: distal urethra), stratified for gender. Outliers are not displayed.

24h-baseline UPT according to the BLD was 1.3mL/min (0.6–3.3mL/min) and 1.1mL/min (0.6–6.3mL/min) in females and males, respectively. DT1-baseline UPT according to the BLD was 2.1mL/min (1.0–4.1mL/min) and 1.8mL/min (0.9–9.7mL/min) in females and males, respectively. The median specific urine weight prior to the start of the measurement was 1.010g/mL (1.000–1.030g/mL). The median time used for a stimulation cycle was 957s (525-2623s) with a median produced volume of 90mL (0-670mL) leading to a UPT of 7.2mL/min (0.4–22.7mL/min) and 3.9mL/min (0–25.6mL/min) in females and males, respectively. The values of produced volume during a stimulation cycle are shown in Fig 4A.

The produced urine volume divided by the duration of the whole stimulation cycle was in females and males 5.2 (0.2–19.2) and 3.5 (0–36.5) times higher compared to baseline (V = 142000, p<0.001), respectively (gender-difference: U = 26417, p<0.001). When assuming increased UPT during D_stim_ and analysing what is produced during D_stim_ in addition to baseline, we calculated a median UPT of 9.4mL/min (-2.3–37.8mL/min) in females and 5.4mL/min (-3.1–54.4mL/min) in males, respectively (gender difference: U = 43964, p<0.001). UPT was significantly higher compared to baseline (z = 18.637, p<0.001, n = 89), independently if the baseline from the BLDs was calculated across 24 hours, daytime only (DT1, z = 14.661, p<0.001, n = 89) or daytime volumes minus the first morning urine volume (DT2, z = 17.219, p<0.001, n = 89).

LMM showed that stimulation frequency (p<0.001), stimulation order (p = 0.003), and STIMINT (p = 0.042) had a significant influence on UPT-ratio. Additionally, UPT-ratio was different between genders (p = 0.047), while stimulation location, visit, and age had no significant impact (Table 2).

**Table 2:** Linear mixed effect model showing fixed and random effects on urine production per time-ratio.

Name		Estimate	SE	t-value	DF	p-value	Confidence interval (95%)		Simulated LRT
							Lower	Upper	p-value
**Fixed effects**									
(Intercept)		3.096	4.110	0.753	527	0.452	-4.977	11.169	
Stimulation frequency^a^		4.651	0.564	8.240	527	<0.001	3.542	5.760	<0.001*
Stimulation intensity^b^		0.086	0.040	2.151	527	0.032	0.007	0.165	0.042*
Location^c^									0.230
	*bladder dome*	-1.523	1.677	-0.908	527	0.364	-4.818	1.772	
	*proximal urethra*	0.267	1.657	0.161	527	0.872	-2.987	3.522	
	*membranous urethra*	-1.302	2.116	-0.615	527	0.539	-5.458	2.854	
	*distal urethra*	-3.448	1.659	-2.079	527	0.038	-6.708	-0.189	
Stimulation order^d^									0.003*
	*2nd stimulation*	-1.614	0.605	-2.666	527	0.008	-2.803	-0.424	
	*3rd stimulation*	-2.060	0.617	-3.338	527	0.001	-3.273	-0.848	
Age^e^		0.118	0.154	0.767	527	0.443	-0.185	0.422	0.462
Gender^f^		-2.529	1.201	-2.105	527	0.036	-4.889	-0.169	0.047*
Visit^g^		0.060	0.507	0.119	527	0.905	-0.936	1.056	0.901
**Random effects**									
Group		Name	SD						
Subject		(Intercept)	4.671						
Residual			5.702						
n	90								
Adjusted R^2^	0.453								

DF: degrees of freedom; n: number of subjects; SD: standard deviation; SE: standard error; Simulated LRT: simulated likelihood ratio test

^a^Baseline = 0Hz

^b^Baseline = 0mA

^c^Baseline = Trigone

^d^Baseline = first stimulation

^e^Baseline = 0 years

^f^Baseline = females

^g^Baseline = Visit 1.

Asterisk (*) indicates statistical significance p<0.05.

**Fig 5:**
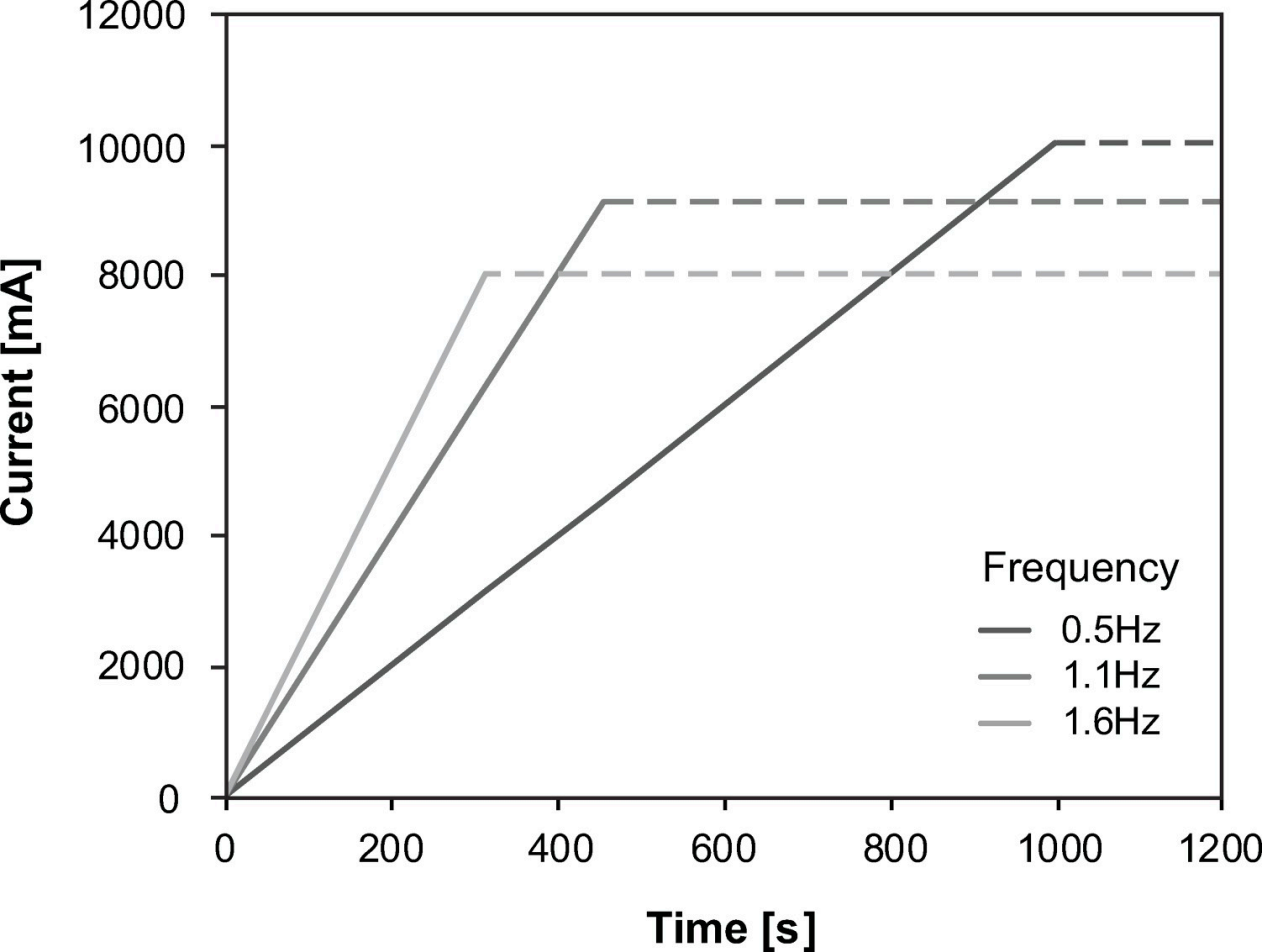
Accumulated current output per time across 500 stimuli for the three stimulation frequencies. The accumulated current was calculated based on mean absolute stimulation intensity (STIMINT) across all stimulation locations.

For frequency, a positive linear increase of UPT-ratio was observed (estimate = 4.651/Hz, Table 2, Fig 4B). LMM demonstrated that an increase of STIMINT by 1mA would lead to a rise of UPT-ratio of 0.086 (Table 2). STIMINTs were greater when stimulating with lower frequencies (0.5Hz: 16.8mA (3.6–74.0mA), 1.1Hz: 14.40mA (4.4–63.4mA), 1.6Hz: 13.6mA (3.6–57.8mA)). Fig 5 shows applied current per time for the different frequencies.

Stimulation location had no significant influence on UPT-ratio, however pairwise comparisons revealed higher UPT-ratio during stimulation at TG compared to dUR (estimate = -3.448, p = 0.038). For the first stimulation cycle, LMM showed that the increase in UPT was higher compared to the second (estimate = -1.614, p = 0.008) and third stimulation cycle (estimate = -2.060, p = 0.001). Additionally, females showed a higher UPT-ratio compared to males during LUT stimulation (estimate = -2.529, p = 0.036, Table 2). Supplementary LMMs (S2–S6 Tables) revealed similar results for fixed effects stimulation frequency, stimulation order, gender (not significant when using DT2 baseline values) and STIMINT (for LMM excluding mUR and for LMM including DT1- and DT2-baseline values).

## Discussion

This is the first study investigating the relationship between LUT electrical stimulation and urine production. Electrical stimulation significantly increased UPT compared to baseline BLD values. The urine production during electrical stimulation increased to such an extent that even considering baseline BLD values from daytime only (DT1) still resulted in a 3.3 fold increase in UPT.

According to our hypothesis, higher frequencies had a bigger impact on UPT-ratio. By definition, higher frequencies cause a higher accumulated current output per time when the pulse width (1ms) remains constant. This is confirmed by our results even when considering the differences in STIMINT (Fig 5). We assume that application of a higher accumulated current per time to the LUT afferent nerves has an enhanced effect on the UPT-ratio, which in turn might also explain the significant, although smaller effect of STIMINT on urine production. However, the exact role of STIMINT requires further elucidation as the significant effect disappeared when excluding outliers or taking produced volume as outcome measure. Likewise, the mechanism underlying the observed frequency effect needs further investigation.

While visit had no significant impact on UPT-ratio, the biggest increase was observed during the first stimulation with a subsequent decrease over time and stimulations. Rather than habituation effects we suggest homeostatic reasons responsible for this continuous decrease in UPT-ratio over time. Although the simulated LRT did not reveal a significant overall effect of location on UPT-ratio, the exploratory pairwise comparisons showed significantly higher impact of the location TG compared to dUR (p = 0.038). This result has to be interpreted with caution and further investigations are needed in larger sample sizes. A higher increase for TG would correspond well with a 3-dimensional image reconstruction study reporting that autonomic innervation is predominant at the bladder neck in females and males [8]. This interpretation would be supported by histochemical-/ electron microscopy studies showing a higher fiber density in the regions of the TG [13, 14]. However, there remains controversy about the topographical distribution of human LUT innervation.

Our observation of increased UPT during LUT stimulation is a relevant finding from a physiological perspective but also for diagnostic and therapeutic purposes in the context of cardiovascular and diuretic dysregulation. Several possibilities of intravesical electrical stimulation for the treatment of bladder dysfunction were previously reported. It was used to cause detrusor contractions or to modulate activity of neuronal pathways [15–17]. Nevertheless, according to the literature there is no concept of knowledge on the functional interrelation of LUT electrical stimulation and renal urine production.

The described effect might be based on altered renal sympathetic activity due to vagal stimulation on LUT level. It was reported in animal studies that vagal afferent stimulation leads to frequency-dependent reductions in renal sympathetic nerve activity, renal release of dopamine and natriuresis [18, 19]. In humans, central and peripheral inputs to the brain (i.e. nucleus tractus solitarius, caudal and rostral ventrolateral medulla) regulate efferent renal sympathetic nerve activity including the somatosensory and viscerosensory systems [20]. However, it is still not known whether this input is excitatory or inhibitory [20].

Despite our efforts the subjects were probably exposed to a certain level of stress and anxiety. However, psychological stress due to anxiety and shame would lead to increased sympathetic activity which based on our hypothesis would rather decrease or at least not increase urine production [21, 22]. The amount of urine production derived from the BLDs was comparable between genders and to values reported in the literature [23, 24]. However, during electrical LUT stimulation UPT-ratio was larger in females than males. This gender difference during stimulation could be caused by differing autonomic modulation, i.e. by a more pronounced parasympathetic tone or susceptibility reported in females [25, 26]. This could lead to a stronger inhibition of the sympathetic renal nerves during LUT stimulation and thereby increase UPT. Otherwise, a shorter duration of the different sections (D_cath_, D_thr_, D_empt_) of each stimulation cycle in females could explain to some extent the gender difference in UPT-ratio. Gender effects should be interpreted with caution considering that there was no gender effect anymore when reanalysing the data including only daytime BLD values minus the first morning urine volume (DT2).

Understanding such interrelations might be relevant for patients with impaired urine production, such as patients with kidney or heart failure, and for methodological aspects in the assessment of LUT afferent function. Despite the low risk of this intervention, further investigations are necessary in patients to evaluate its feasibility and therapeutic value. Further studies investigating age effects, possible confounders (i.e. catheter and contrast agent) and including assessments of the autonomic nervous system (i.e. blood pressure, heart rate, sympathetic skin response and renal resistance index) and urine osmolarity are mandatory for a further understanding of the involved mechanisms and the neurophysiological interactions between the lower and upper urinary tract.

### Limitations

Limitations of this study are 1) lack of volume measurements for each section (D_cath_-D_empt_) of the stimulation cycle to better differentiate the contribution of each section to the observed effect. 2) Baseline UPT during the measurement was possibly slightly underestimated since the catheter or contrast agent could lead to certain UPT increase, but this would not explain the strong frequency effect.

## Conclusions

There was a clear effect of LUT electrical stimulation on UPT shown in healthy subjects with a greater impact of higher frequencies. This might not only be relevant for methodological aspects in the assessment of LUT afferent function but also for patients with impaired urine production. The mechanisms behind our findings are still unclear warranting further investigations to confirm validity and to find physiological explanations for the mechanism of action.

## Supporting information

S1 Checklist CONSORT 2010 checklist for randomized trials. (DOC)

S1 Study Protocol Approved protocol by the local ethics commission. (PDF)

S1 Fig Box plots of median, 25th and 75th percentile and whiskers of individual stimulation sections. D_cath_ [s] (S1a) D_thr_ [s] (S1b), and D_empt_ [s] (S1c) for the three stimulation frequencies and five stimulation sites, stratified for gender. Outliers are not displayed. D_cath_ = time [s] used for catheter positioning at specific stimulation location; D_thr_ = time [s] used for current perception threshold (CPT) / pain threshold assessment and definition of absolute stimulation intensity (STIMINT); D_empt_ = time [s] used for bladder emptying.
(TIF)

S1 TableBaseline characteristics stratified for stimulation location and gender.Locations: Bladder dome (BD, S1a), trigone (TG, S1b), proximal urethra (pUR, S1c), membranous urethra (mUR, S1d), distal urethra (dUR, S1e). Data are represented as (^a^) mean±standard deviation (SD), (^b^) median (range: minimum-maximum) or number of subjects (n) if appropriate. All subjects fulfilled predefined cut-offs for study inclusion: MoCA score ≥26, HADS ≤7 each, IPSS ≤7, BLD: $\frac{24h\;urinary\;frequency}{drinking\;volume\;[mL]}\leq 0.0045$ with a maximum of 1x nocturia, mean volume per void >150mL and absence of urinary incontinence or urgency. (°) indicates significant gender differences p<0.05. ^+^ due to different scoring systems, female and male subjects have not been compared. ICIQ = International Consultation on Incontinence Modular Questionnaire, FLUTS = Female lower urinary tract symptoms, MLUTS = Male lower urinary tract symptoms, IPSS = International Prostate Symptom Score, OAB-q SF = The Overactive Bladder Questionnaire short-form, QoL = Quality of life, HADS = Hospital Anxiety and Depression Scale, MoCA = Montreal Cognitive Assessment.(DOCX)

S2 Table Linear mixed effect model excluding mild outliers of the urine production per time (UPT)-ratio. DF: degrees of freedom; n: number of subjects; SD: standard deviation; SE: standard error; Simulated LRT: simulated likelihood ratio test; ^a^Baseline = 0Hz; ^b^Baseline = 0mA; ^c^Baseline = Trigone; ^d^Baseline = first stimulation; ^e^Baseline = 0 years; ^f^Baseline = females; ^g^Baseline = Visit 1; Asterisk (*) indicates statistical significance p<0.05.
(DOCX)

S3 Table Linear mixed effect model excluding stimulation location membranous urethra. DF: degrees of freedom; n: number of subjects; SD: standard deviation; SE: standard error; Simulated LRT: simulated likelihood ratio test; ^a^Baseline = 0Hz; ^b^Baseline = 0mA; ^c^Baseline = Trigone; ^d^Baseline = first stimulation; ^e^Baseline = 0 years; ^f^Baseline = females; ^g^Baseline = Visit 1; Asterisk (*) indicates statistical significance p<0.05.
(DOCX)

S4 Table Linear mixed effect model with the produced volume [mL] as outcome measure. DF: degrees of freedom; n: number of subjects; SD: standard deviation; SE: standard error; Simulated LRT: simulated likelihood ratio test; ^a^Baseline = 0Hz; ^b^Baseline = 0mA; ^c^Baseline = Trigone; ^d^Baseline = first stimulation; ^e^Baseline = 0 years; ^f^Baseline = females; ^g^Baseline = Visit 1; Asterisk (*) indicates statistical significance p<0.05.
(DOCX)

S5 Table Linear mixed effect model for urine production per time-ratio when using daytime DT1-baseline. DT1-baseline: all daytime urine volumes from bladder diary; DF: degrees of freedom; n: number of subjects; SD: standard deviation; SE: standard error; Simulated LRT: simulated likelihood ratio test; ^a^Baseline = 0Hz; ^b^Baseline = 0mA; ^c^Baseline = Trigone; ^d^Baseline = first stimulation; ^e^Baseline = 0 years; ^f^Baseline = females; ^g^Baseline = Visit 1; Asterisk (*) indicates statistical significance p<0.05.
(DOCX)

S6 Table Linear mixed effect model for urine production per time-ratio using daytime DT2-baseline. DT2-baseline: daytime urine volumes from bladder diary (BLD) minus the first morning urine volume; DF: degrees of freedom; n: number of subjects; SD: standard deviation; SE: standard error; Simulated LRT: simulated likelihood ratio test; ^a^Baseline = 0Hz; ^b^Baseline = 0mA; ^c^Baseline = Trigone; ^d^Baseline = first stimulation; ^e^Baseline = 0 years; ^f^Baseline = females; ^g^Baseline = Visit 1; Asterisk (*) indicates statistical significance p<0.05.
(DOCX)
