# Supplementary material for: Does electrical stimulation in the lower urinary tract increase urine production? A randomised comparative proof-of-concept study in healthy volunteers
Source: PLoS One. 2019 May 24;14(5):e0217503. doi: 10.1371/journal.pone.0217503 (PMC6534346; doi:10.1371/journal.pone.0217503)
Supplement: S1 Study Protocol — (PDF) [file pone.0217503.s002.pdf]

Study Protocol

1 TITLE

Study and protocol title:

Evaluation of new approaches in the treatment and neurophysiological assessment of healthy and impaired human lower urinary tract function (SENSORY II)

version date: 08.09.2015

1.1 PRINCIPLE INVESTIGATORS

**Dr. med. Ulrich Mehnert**<br>Neuro-Urology<br>Spinal Cord Injury Center & Research<br>University of Zürich<br>Balgrist University Hospital<br>Forchstrasse 340<br>8008 Zürich<br>Switzerland	
Signature	

1.2 LIST OF EMPLOYEES

**PD Dr. med. Thomas M. Kessler**<br>Neuro-Urology<br>Spinal Cord Injury Center & Research<br>University of Zürich<br>Balgrist University Hospital<br>Forchstrasse 340<br>8008 Zürich<br>Switzerland	**Dr. med. Marc Schneider**<br>Neuro-Urology<br>Spinal Cord Injury Center & Research<br>University of Zürich<br>Balgrist University Hospital<br>Forchstrasse 340<br>8008 Zürich<br>Switzerland
**Dr. phil. Martina D. Liechti**<br>Neuro-Urology<br>Spinal Cord Injury Center & Research<br>University of Zürich<br>Balgrist University Hospital<br>Forchstrasse 340<br>8008 Zürich<br>Switzerland	**Stephanie van der Lely**<br>Neuro-Urology<br>Spinal Cord Injury Center & Research<br>University of Zürich<br>Balgrist University Hospital<br>Forchstrasse 340<br>8008 Zürich<br>Switzerland
**Pract. med. Martina Stefanovic**<br>Neuro-Urology<br>Spinal Cord Injury Center & Research<br>University of Zürich<br>Balgrist University Hospital<br>Forchstrasse 340<br>8008 Zürich<br>Switzerland	**Dr. med. Stephanie Knüpfer**<br>Neuro-Urology<br>Spinal Cord Injury Center & Research<br>University of Zürich<br>Balgrist University Hospital<br>Forchstrasse 340<br>8008 Zürich<br>Switzerland

**Melanie Schmidhalter**<br>**Neuro-Urology**<br>Spinal Cord Injury Center & Research<br>University of Zürich<br>Balgrist University Hospital<br>Forchstrasse 340<br>8008 Zürich<br>Switzerland	

2 OBJECTIVES AND PURPOSE

2.1 BACKGROUND AND OBJECTIVE OF THE STUDY

Lower urinary tract symptoms (LUTS) as urinary urgency, frequency, and incontinence have great impact on the health-related quality of life, including impairments in sexuality, emotional well-being and productivity at home and at work [1, 2]. The estimated worldwide prevalence of LUTS is high with 45% having at least one LUTS [3]. Consequently, there is an enormous economic burden for every health care system [4, 5].
A large proportion of LUTS (i.e. overactive bladder (OAB)) affects the storage phase and is attributed to aberrant sensory function of the LUT [1, 3, 6, 7]. However, in many cases the exact causes and pathologic mechanisms responsible for LUTS are unknown, which might be partly due to a lack of accurate and specific diagnostic tools. There is currently no objective and reliable clinical assessment tool of human bladder and urethral afferent nerve function and integrity available. Such an assessment tool would enable a greater understanding of the role of sensory nerves in LUTS.
So far, clinical assessment of bladder sensations relies on filling cystometry (FC). During this urodynamic examination, patients usually indicate a first sensation of filling (FSF), a first desire to void (FDV) and a strong desire to void (SDV), and in pathologic cases urgency. Although this gives important information about the character of bladder sensations, it is not an objective measurement because it relies on the compliance, alertness, and subjective feelings of the patient. Thus, the objectivity and reliability of FC is discussed controversial: There are studies questioning the reliability of FC, in which only few urodynamic parameters show excellent reliability, others in turn affirm it [8-11]. Committee No. 25 of the International Consultation on Incontinence (ICI) states that there is limited and inadequate information regarding the reproducibility, accuracy, sensitivity, specificity, and the predictive value of urodynamic tests [12]. Therefore, there is an ongoing quest for an objective and reliable method to improve evaluation of bladder sensations in humans. Committee No. 7 of the ICI strongly recommends such an improvement of diagnostic performance with regard to its objectivity and reproducibility [13]. In addition, FC does only reflect sensory information from the bladder but not from the urethra.
To improve sensory assessment of the LUT several groups have investigated current perception threshold (CPT) testing at different sites of the LUT including the urethra. First reports on LUT CPTs go back to 1899 using quite invasive setups with monopolar stimulation [14]. Recent studies used mainly bipolar stimulation to provide more organ specific CPTs as bipolar stimulation enables a more localized current that limits the stimulation to the bladder afferent nerves [15].
In patients with neurogenic lesions or diseases (i.e. diabetic neuropathy, Parkinson’s disease, multiple sclerosis, spinal cord lesion) expectedly higher bladder CPTs were observed compared to healthy subjects [16-18]. Generally, higher values have been reported for bladder CPTs than for urethral CPTs [19-23] and ageing seems to be associated with higher urethral CPTs [22, 24]. Pelvic surgery has been reported to increase bladder [22] and urethral CPTs [25, 26]. However, some results are conflicting regarding urethral CPTs following pelvic surgery [22, 25, 26], which might be attributed to the type and radicalness of surgery.
Urgency urinary incontinence patients demonstrated significantly higher urethral CPTs compared to controls [24], which could be partly “normalized” by oral antimuscarinics [27]. Conflicting results have been reported in non-neurogenic OAB (NNOAB) patients, with significantly lower bladder CPTs [28] but also equal bladder CPTs compared to non-OAB/healthy subjects [15, 24].

In patients with neurogenic DO (NDO) [29] and NNOAB [30] it was demonstrated that antimuscarinic treatment significantly elevated CPTs which was interpreted as evidence that antimuscarinic drugs affect LUT sensory function, which is in line with animal studies and previous clinical studies in humans [31-33].
Elaboration on methodological aspects revealed that next to pulse frequency and duration, electrode position and bladder volume have a significant effect on the CPT results (sensitivity of the bladder can be increased by filling the bladder) and should thus be mentioned when reporting CPT outcomes from the LUT and standardized if possible [34]. However, only few studies report on these important aspects and a huge variety of CPT parameters and techniques have been used which makes a valid comparison difficult.
Several, more recent studies started to use a commercially available automated CPT measurement system called “Neurometer®” (Neurotron Inc., Baltimore, USA) that is claimed to be able to

differentiate between CPTs of C-fibers, A-delta fibers, and A-beta fibers using sine wave frequencies of 5Hz, 250Hz, and 2000Hz, presumably capable of selective activation of each of these fiber types respectively [16, 21, 24]. Although the principle of frequency-related neuroselectivity due to the different fiber specific refractory periods in relation to their fiber diameter appears theoretically plausible, there are doubts that this approach is readily transferable to CPT measurements in the human LUT. One of the articles that is frequently cited in Neurometer® studies as proof of neuroselectivity is a neurophysiological study in rats demonstrating a certain selectivity of A-beta, A-delta, and C-fibers in response to 2000Hz, 250Hz, and 5Hz stimulation respectively [35]. However, only 2000Hz was demonstrated to be purely A-beta fiber specific, but 250Hz and 5Hz activated both, A-delta and A-beta fibers, and C-fibers and A-delta fibers, respectively [35]. Thus it remains still necessary to clarify how specific and sensitive neuroselective CPT measurements can be performed in the LUT. The skepticism is supported by a study investigating LUT CPTs using the Neurometer® in patients with idiopathic DO, lacking to demonstrate a significant difference in 5Hz CPTs before and after resiniferatoxin instillation into the bladder [36], although resiniferatoxin is known to desensitize bladder afferent C-fibers [7, 36]. Another issue that needs further clarification is the presence of A-beta fibers in the LUT. Studies using the Neurometer® for LUT CPT measurement persistently present CPT values after 2000Hz stimulation of the bladder mucosa which would indicate the presence of A-beta fibers in the LUT [16, 21, 24, 30]. However, A-beta fibers have never been described in the LUT [37].

Although CPTs are believed to reflect the level of responsiveness of the afferent nerves, this method still remains semi-objective and no correlation between urinary symptoms, cystometric filling sensations and LUT CPTs could be found neither with square nor sine wave stimulation [21, 38], whereas sine wave stimulations seems to result in less reliable CPTs compared to square waves [15].

Other, somewhat older studies investigated the possibility to record sensory evoked potentials (SEPs) following LUT electrical stimulation. The clinical utility of SEPs is based on their ability to reveal abnormal sensory function, when findings from previous investigations (i.e. history, neurologic examination, urodynamic examination) are equivocal [39].

Through analysis of latencies and amplitudes, SEPs provide objective information of nerve fiber integrity and in relation to the conduction velocity also on the fiber type.

The previous studies presented first feasibility results of SEP recording from the LUT following electrical stimulation of the vesico-urethral junction and the posterior urethra in healthy subjects [40-42, 67] and patients [43-46]. In these studies, mainly bipolar stimulation was used, as monopolar stimulation is less LUT specific due to the high probability to impinge on several electrically excitable structures between the cathode (intravesical) and anode (on the surface of thigh or abdomen).

Although most stimulations were performed with 2Hz at similar sites, the heterogeneous study populations and inconsistencies of some important measurement settings (i.e. bladder volume, stimulation strength and recording filter parameters) hamper a meaningful comparison of those studies and resulted in varying SEP shapes and latencies. Unfortunately, this approach was not systematically followed up until now and there is a lack on studies investigating the reliability of this method, providing normative data and data from other LUT sites (i.e. trigone, bladder dome), and exploring methodological standards.

There is a lack of an objective marker for LUT afferent function and a subtle instrument to distinguish between true neurogenic LUT dysfunction and non-neurogenic, end organ related (i.e. urothelial derived OAB) LUT dysfunction.

Based on our previous study, LUT SEPs have the potential to be such an objective marker [67]. We now aim to validate our method as an objective marker for clinical diagnostics that allows the evaluation of pathological LUT conditions and its distinction from healthy LUT neurophysiology and function.

Furthermore, data from the LUT SEP can readily be compared to urodynamic findings and common SEP data from the legs and pudendal somatosensory afferents. This will allow a detailed neurophysiological workup of each patient to classify expected changes in the LUT afferent system.

In addition, we aim to evaluate the use of LUT SEPs as an instrument to objectively assess the effect of LUT therapies targeting its afferents (i.e. botulinum toxin, antimuscarinics, sacral neuromodulation). LUT SEPs might essentially contribute to a better understanding of the mechanism of action of those LUT therapies.

Objectives:

- To advance the evaluation of viscerosensory afferent pathways in healthy subjects and patients with lower urinary tract symptoms (LUTS) or dysfunctions using SEPs to promote the

understanding of afferent alterations leading to LUT symptoms and to advance a more precise assessment of LUT function.

- To refine the methodology of LUT SEPs
- To investigate the differences in LUT sensory perception between healthy subjects and patients with LUT dysfunction
- To investigate the effect of established and approved therapies*

  *LUTS therapies will be applied completely independent from the study and their application relies solely on the decision of the currently treating physician, e.g. urologist/gynaecologist, who is not involved in this study.

2.2 QUESTIONS, TRIAL POPULATION

The study consists of two parts with different main focus/questions and study population.

<u>Part I:</u>
a) What is the influence of different stimulation parameters (e.g. stimulation frequency) on the reliability, shape, latency, amplitude, and topographical distribution of SEPs recorded during electrical stimulation of the LUT?
b) Is there any potential effect of subject age and sex on LUT SEPs?
c) Does the desire to void and corresponding bladder filling volume have an impact on the latencies and amplitudes of LUT SEPs?
d) Is there a correlation between LUT SEPs and outcome parameters from other neurophysiological, e.g. tibial, pudendal, and S3 dermatome sensory evoked potentials (SSEPs) and contact heat evoked potentials (CHEPS), and neuro-urological, e.g. urodynamics, assessments.

Trial population: Healthy adult volunteers (n = at least 120, females and males, age >18 years).

<u>Part II:</u>
a) Does the assessment of LUT SEPs provide reliable markers for LUTS?
b) Is there any relation between LUT SEP measures and the symptom severity (recorded with standardized questionnaires) or urodynamic outcomes in patients with LUTS?
c) Do SEP measures allow assessment of potential treatment effects of established and approved LUTS therapies?
d) Is there a correlation between LUT SEPs and outcome parameters from other neurophysiological, e.g. tibial, pudendal, and S3 dermatome SSEPs and CHEPS, and neuro-urological, e.g. urodynamics, assessments.

Trial population: Patients (n=100, females and males, age >18 years) with neurogenic and non-neurogenic LUTS.

2.3 HYPOTHESIS

Part I:

- Effectiveness of the stimulation frequency, to obtain an SEP will be related to the prevailing afferent conduction velocity due to fiber composition at the stimulation localization and it can be increased up to the reciprocal value corresponding to this conduction velocity without compromising SEP recording.
- Higher frequencies decrease SEP return rates due to fiber refractoriness
- Aging will increase current perception thresholds (CPTs) and decrease latencies of LUT SEPs.

Part II:

- Patients with LUTS will show significantly decreased latencies and pathological LUT SEPs compared to healthy subjects.
- LUT SEPs recorded at SDV will show shorter latencies due to a facilitating effect of bladder distention.

- LUT SEP latencies will correlate negatively with urgency severity and maximum cystometric capacity.
- Treatment for LUTS will significantly decrease LUT sensory thresholds and LUT SEPs amplitudes but not latencies.

3 DESIGN OF THE STUDY

3.1 TARGETED PRIMARY AND SECONDARY ENDPOINTS

**Primary endpoint**	N1 responder rate / latency of LUT SEPs
**Secondary endpoints**	Latencies (P1, P2), amplitudes (P1, N1, P2, P1N1-, N1P2), topographies and source localizations of LUT SEPs;<br>Latencies, amplitudes, topographies and source localisations of tibial, pudendal, and S3 dermatome SSEPs and CHEPS;<br>Current perception thresholds (CPTs), 3 day bladder diary, scores of ICIQ-FLUTS/MLUTS, IPSS, IIEF/FSFI, Swiss German OAB, ICIQ-LUTSqol, Qualiveen, HADS, MoCA, urodynamic parameters (such as maximum cystometric capacity, bladder volume at FSF, FDV, and SDV, maximum detrusor pressure during storage phase, bladder compliance, detrusor overactivity, maximum flow rate, voided volume, post void residual, maximum detrusor pressure during voiding phase, detrusor pressure at maximum flow rate, pelvic floor electromyography).

3.2 STUDY DESIGN

**<u>Study type:</u>**	Prospective, investigator initiated, non-randomized, single center, clinical basic research study
**<u>Study site:</u>**	Neuro-Urology, Spinal Cord Injury Center & Research, University of Zurich, Balgrist University Hospital

The project will consist of two parts:

<u>Part I:</u> LUT SEP measurements in healthy subjects (Figure 1 and 2). Part I consists of 3 visits, screening (Visit 1), study measurement one (Visit 2), and study measurement two (Visit 3).

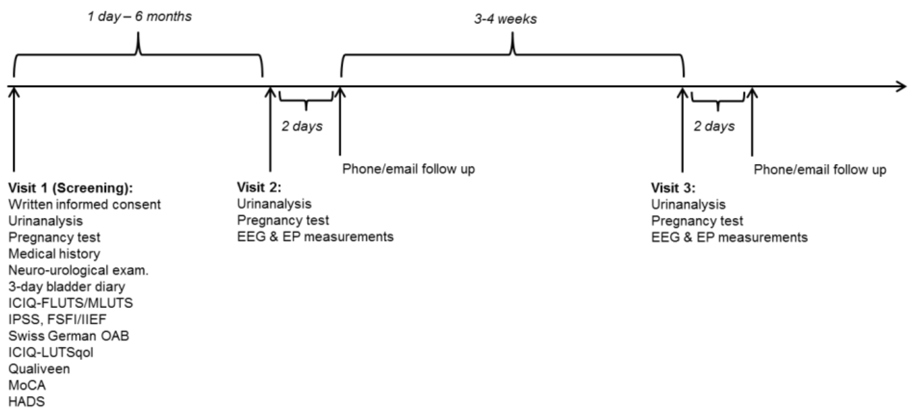

Figure 1: Sequence of visits and assessments for healthy volunteers during study part I. ICIQ-FLUTS/MLUTS International Consultation on Incontinence Questionnaires for male or female LUTS, IPSS International Prostate Symptom Score, IIEF/FSFI International Index of Erectile Function/Female Sexual Function Index, Swiss German OAB Swiss German Overactive Bladder questionnaire, ICIQ-LUTSqol Lower Urinary Tract Symptoms Quality of Life, HADS Hospital Anxiety and Depression Scale, MoCA Montreal Cognitive Assessment, EEG electroencephalogram, EP evoked potential

Screening (Visit 1, 1-2 hours): First, volunteers are informed about the entire study, the planned investigations, and the required behavior. After having provided written informed consent, they will be screened for their medical history, current medication, vital signs and their neurological status. Urine will be analyzed to exclude pregnancy or urinary tract infection. Subsequently a neuro-urological examination will be performed. All subjects are required to complete a 3-day bladder diary (including pad test), the International Consultation on Incontinence Questionnaires for male or female LUTS (ICIQ-FLUTS/MLUTS), the International Prostate Symptom Score (IPSS), the International Index of Erectile Function/Female Sexual Function Index (IIEF/FSFI), the Overactive Bladder questionnaire (SwissGerman OAB), the International Consultation on Incontinence Questionnaire for lower urinary tract symptoms quality of life (ICIQ-LUTSqol), the Qualiveen quality of life measure for individuals with urinary disorders, the Hospital Anxiety and Depression scale (HADS) and the Montreal Cognitive Assessment (MoCA).

Study measurement 1 and 2 (Visits 2 and 3, each 3 hours): Prior to each measurement, urine will be analyzed to exclude pregnancy or urinary tract infection. Each session consists of a resting EEG measurement followed by recordings of cortical evoked potentials elicited by transurethral electrical LUT stimulation (LUT SEPs) and transcutaneous electrical (SSEPs) and heat stimulations (CHEPS) at different body sites. A detailed sequence of measurements during visits 2 and 3 is summarized in Figure 2.
Each EEG session comprises several neurophysiological standard assessments, including recordings of the electrooculogram (left and right eye), electrocardiogram, electromyogram andelectroencephalogram (EEG) using a 64 Ag/AgCl surface electrodes system comprising a cap-based extended international 10-20 montage (Easy cap, Easy cap GmbH, Munich, Germany). Electrode impedances will be constantly kept below 20kΩ. In addition, six needle electrodes will be placed in the scalp, respectively above the spine and the iliac crest for routine clinical SEP recording for a segmental assessment.
Intravesical stimulation will be applied transurethrally using a custom made 14 Ch catheter (Unisensor AG, Attikon, Switzerland) comprising three platinum electrodes and a radiopaque marker, which allows precise catheter positioning under fluoroscopic control.The same catheter allows to control bladder volumes during LUT stimulation. After each intravesical stimulation the bladder will be drained and refilled with the same amount of contrast medium (Ultravist 150, Bayer Schweiz AG, Switzerland) to ensure constant measurement conditions. For intravesical stimulation, electrical pulses will be applied repeatedly at different LUT sites using different stimulation parameters in terms of stimulation frequency (0.1-5Hz), pulse shape (monopolar, bipolar, rectangular and sinusoidal) and pulse width (0.1-1ms). Stimulation intensity will be individually adapted to produce clear but tolerable sensation, which typically is 3-4 times sensory threshold. All LUT stimulations will be performed at low bladder volumes, e.g. 0-100ml, and high bladder volumes, e.g. volume at SDV.

In addition, standard neurophysiological measurements such as tibial, pudendal, and S3 SSEPs and CHEPS will be performed for subsequent comparison with LUT SEPs.

Follow-up: Two to three days after each visit, a follow-up interview via telephone or e-mail questionnaire will be performed to evaluate the general wellbeing of the subjects and to exclude any adverse effects (Figure 1). Concerning the telephone and e-mail follow up we will use a standardized questionnaire (see also separate follow up form). In case of adverse events, subjects will be appointed to an extra visit for further evaluation and treatment if applicable.

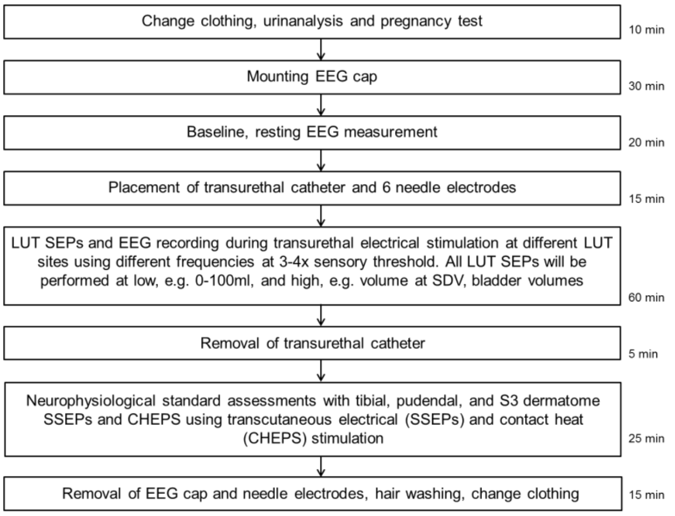

Figure 2: Sequence of procedures during study measurements 1 and 2 (Visits 2 and 3) in healthy subjects.

<u>Part II:</u> LUT SEP measurements in patients with LUTS (Figure 3 and 4). Part II consists of 4 visits, screening (Visit 1), study measurement one (Visit 2), study measurement two (Visit 3) and eventually a third post-treatment measurement (Visit 4) (Figure 3 and 4).

Screening (Visit 1, 1 hour, Figure 3): First, patients are informed about the entire study, the planned investigations, and the required behavior. After having provided written informed consent, they will be screened for their medical history, current medication, vital signs and their neurological status. Urine will be analyzed to exclude pregnancy or urinary tract infection. Subsequently a neuro-urological examination will be performed. All patients are required to complete a 3-day bladder diary (including pad test), the International Consultation on Incontinence Questionnaires for male or female LUTS (ICIQ-FLUTS/MLUTS), the International Prostate Symptom Score (IPPS) and the International Index of Erectile Function/Female Sexual Function Index (IIEF/FSFI) , the SwissGerman OAB, the ICIQ-LUTSqol, the Qualiveen, the HADS and the MoCA.

Study measurements 1 and 2 (Visits 2 and 3, each 3 hours): Prior to each measurement, urine will be analyzed to exclude urinary tract infection or pregnancy.
In the case that a LUT medication has been stopped at Visit 1 (for instance stopped antimuscarinic treatment, stopped alpha-blocker treatment etc.), a 3-day bladder diary (including pad test), the International Consultation on Incontinence Questionnaires for male or female LUTS (ICIQ-FLUTS/MLUTS), the International Prostate Symptom Score (IPSS), the International Index of Erectile Function/Female Sexual Function Index (IIEF/FSFI), the SwissGerman OAB, the ICIQ-LUTSqol, and the Qualiveen will be completed again 2 weeks after washout of the LUT relevant medication to evaluate any changes in LUTS.
The study measurements 1 and 2 of part II are equally structured to the study measurements 1 and 2 (Visit 2 and 3) of part I (Figure 2).

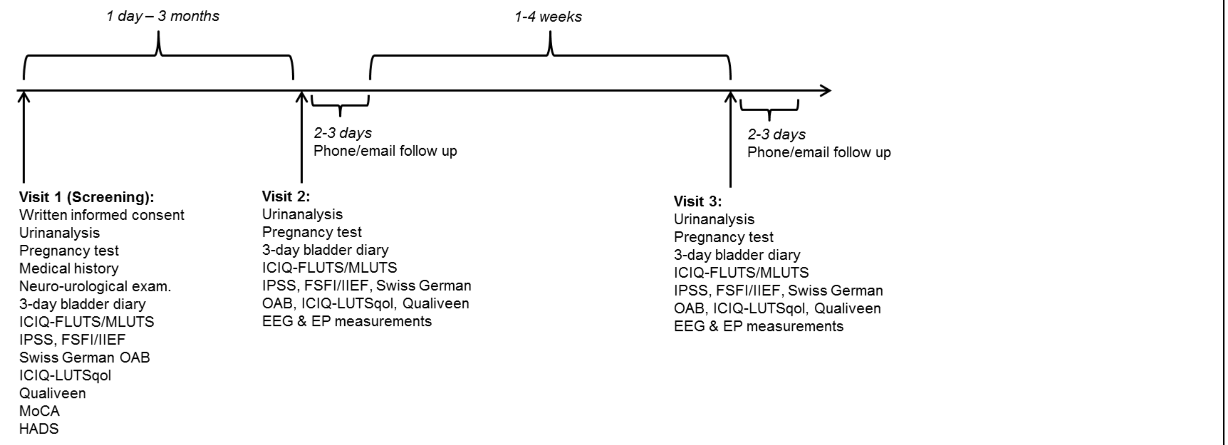

Figure 3: Sequence of visits and assessments for patients with LUTS during study part II. ICIQ-FLUTS/MLUTS International Consultation on Incontinence Questionnaires for male or female LUTS, IPSS International Prostate Symptom Score, IIEF/FSFI International Index of Erectile Function/Female Sexual Function Index, SwissGerman OAB Swiss German Overactive Bladder questionnaire, ICIQ-LUTSqol Lower Urinary Tract Symptoms Quality of Life, HADS Hospital Anxiety and Depression Scale, MoCA Montreal Cognitive Assessment, EEG electroencephalogram, EP evoked potential

Post-treatment measurement 3 (Visit 4, 3 hours, Figure 4): In case of an independent treatment for LUTS, patients are invited for a post-treatment visit. After stated treatment prior to post-treatment measurement, urine will be analyzed to exclude urinary tract infection or pregnancy. Preparation, organization and implementation complies equally to measurement 1 and 2 of part 1. Complete 3-day bladder diary (including pad test), the International Consultation on Incontinence Questionnaires for male or female LUTS (ICIQ-FLUTS/MLUTS), the International Prostate Symptom Score (IPPS) and the International Index of Erectile Function/Female Sexual Function Index (IIEF/FSFI), the SwissGerman OAB, the ICIQ- LUTSqol, and the Qualiveen provide information about effectiveness of medical treatment related to LUTS. Two days after measurement we will use a standardized questionnaire concerning follow up (Figure 4).

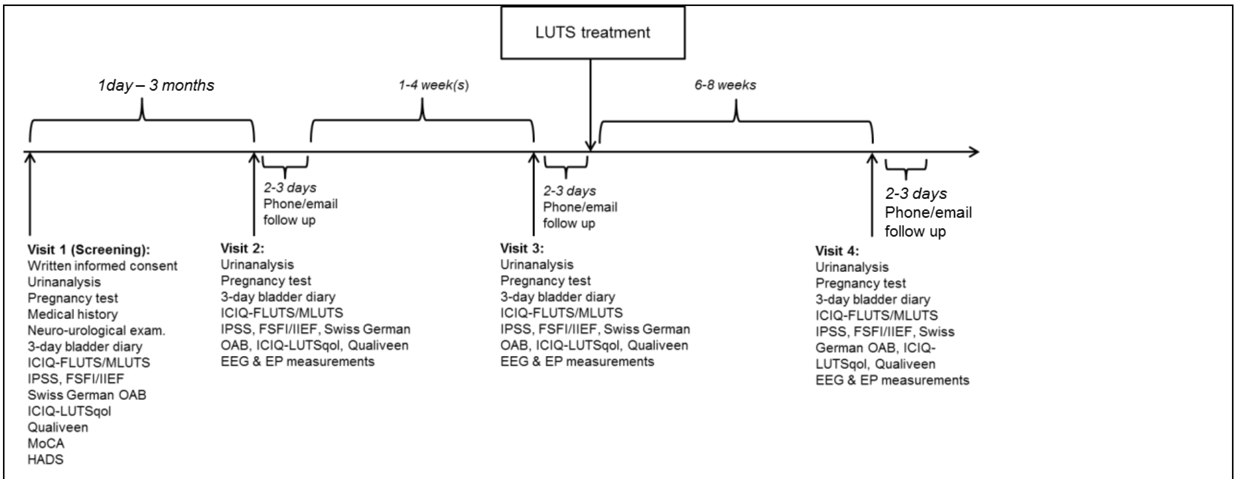

Figure 4: Sequence of visits and assessments for patients with LUTS receiving study independent LUTS treatment during study part II. ICIQ-FLUTS/MLUTS International Consultation on Incontinence Questionnaires for male or female LUTS, IPSS International Prostate Symptom Score, IIEF/FSFI International Index of Erectile Function/Female Sexual Function Index, SwissGerman OAB Swiss German Overactive Bladder questionnaire, ICIQ-LUTSqol Lower Urinary Tract Symptoms Quality of Life, HADS Hospital Anxiety and Depression Scale, MoCA Montreal Cognitive Assessment, EEG electroencephalogram, EP evoked potential

Follow-up: Two to three days after each visit, a follow-up interview via telephone or e-mail questionnaire will be performed to evaluate the general wellbeing of the subjects and to exclude any adverse effects (Figure 1). Concerning the telephone and e-mail follow up we will use a standardized questionnaire (see also separate follow up form). In case of adverse events, patients will be appointed to an extra visit for further evaluation and treatment if applicable.

3.3 MEASURE TO MINIMIZE BIAS

Taking into account circadian fluctuations in the EEG, for each individual, the time of the day will be approximately held constant for all EEG measurements. Patients and healthy volunteers will be age matched to minimize an age effect on data interpretation.
The EEG analysis will be conducted on the model of cluster as well as individual analysis to adjudge, if the cluster results depend on individuals or on all measurements.

4 SELECTION OF TRIAL SUBJECTS

4.1 RECRUITMENT

Healthy subjects will be recruited via announcements at the University of Zürich and in local print and online media (i.e. www.marktplatz.ch).
Patients will be recruited via announcements in local print and online media and via patient consultations in the Spinal Cord Injury Center at Balgrist University Hospital, and the Departments of Urology, Gynecology, and Neurology at the University Hospital Zürich. All subjects and patients will be provided with the study information and informed consent form and given sufficient time to ask questions and to consider all of the information provided and the risks associated with participation.

4.2 CRITERIA FOR INCLUSION

Healthy volunteers

- Written informed consent
- Good mental and physical health
- Age >18 years

Patients with LUTS

- Age >18 years
- Written informed consent
- Prior urodynamic investigation
- LUTS since > 6 months (e.g. storage symptoms: ≥ 3 episodes of urinary urgency per week, urinary frequency > 8/24h, determined by a 3-day bladder diary, and/or voiding symptoms: hesitancy, post void residual volume, poor flow) with or without detrusor overactivity (determined by urodynamics)

4.3 CRITERIA FOR EXCLUSION

Healthy volunteers

- Any neurological or urological pathology
- Current pregnancy or lactation
- Urinary tract infection (UTI)
- Hematuria
- Any previous pelvic or spine surgery
- Any craniocerebral surgery
- Any anatomical anomaly of the LUT or genitalia
- Any metabolic disease
- Any LUT malignancy
- Bladder capacity <150 mL or strong desire to void (SDV) already at 60 mL

Patients with LUTS

- Current pregnancy or lactation
- Urinary tract infection (UTI)
- Gross hematuria

5 ASSESSMENT OF EFFECTIVENESS

5.1 EFFECTIVENESS PARAMETERS: MEASURING METHODS AND TIMES

This is a basic research study. Thus, no assessment of effectiveness is implemented.

6 ASSESSMENT OF SAFETY

6.1SAFETY PARAMETERS: MEASURING METHODS AND TIME

Safety parameters before measurements:

- Case history regarding any neurological, urological pathology or any regular medication intake
- Neuro-urological examination of the patients/healthy volunteers
- Urine analysis to exclude UTI. Subjects with a UTI will not undergo the experiment and further microbiological urine analysis will be initiated. If the UTI is symptomatic (fever, dysuria, urgency) empiric oral antibiotic treatment will be started. Otherwise, antibiotic treatment will be applied according to the results of the microbiological urine analysis. The subject can be reassigned to the study, if the microbiological urine analysis shows no evidence of an UTI or the UTI has been successfully treated.
- Pregnancy test. In case of pregnancy, the subject will be excluded from the study and referred to a gynaecologist.

Safety parameters during measurements:

- The research team performing the investigation/measurement and the responsible physician are consistently in interaction with the subjects monitoring vital signs and comfort of the subjects. There is always the opportunity to stop the measurements immediately

Safety parameters after measurements:

- Follow-up by telephone interview or e-mail two days after each measurement to assess general well-being and potential side effects such as dysuria, hematuria, urinary urgency, fever (see also separate follow-up form)

6.2 FOLLOW-UP OBSERVATION FOR STUDY SUBJECTS WITH ADVERSE EVENTS

All adverse events will be followed until they have abated, or until the initial state has been reached or until a stable situation has been reached. Depending on the event, follow-up may require additional tests or medical procedures as indicated, and/or referral to the general physician or a medical specialist.

7 STATISTICS

7.1 DEFINITION OF THE PRIMARY AND SECONDARY ENDPOINTS

**Primary end point**	N1 responder rate / latency of LUT SEPs
**Secondary end points**	Latencies (P1, P2), amplitudes (P1, N1, P2, P1N1-, N1P2), topographies and source localizations of LUT SEPs;<br>Latencies, amplitudes, topographies and source localizations of tibial, pudendal, and S3 dermatome SSEPs and CHEPS;<br>Current perception thresholds (CPTs), 3 day bladder diary, scores of ICIQ-FLUTS/MLUTS, IPSS, IIEF/FSFI, SwissGerman OAB, ICIQ-LUTSqol, Qualiveen, HADS, MoCA, urodynamic parameters (such as maximum cystometric capacity, bladder volume at FSF, FDV, and SDV, maximum detrusor pressure during storage phase, bladder compliance, detrusor overactivity, maximum flow rate, voided volume, post void residual, maximum detrusor pressure during voiding phase, detrusor pressure at maximum flow rate, pelvic floor electromyography).

Covariables: The impact of subject age, gender, and body height on responder rate / latency and amplitude will be evaluated using multivariate analysis as appropriate.

7.2 PLANNED NUMBER OF TRIAL SUBJECTS WITH CLEARLY STATED JUSTIFICATION

We are planning to include at least 120 healthy subjects (study part I) and 100 patients with LUTS (study part II) to realize a meaningful random effects and subgroup analysis.
This is an exploratory basic research study with a pure diagnostic purpose. A power analysis is not applicable. Data will be evaluated on the basis of a case-by-case analysis as well as on cluster analysis.

7.3 DESCRIPTION OF THE STATISTICAL METHODS FORESEEN AND THE PLANNED INTERMEDIATE ASSESSMENTS

We plan to summarize interval scaled variates with medians; means and standard deviations (SD) where appropriate. Dichotomous variates will be described as ratios and percentages.

a) *Univariate analysis*
   We will use t-tests to compare mean between groups and chi-square/Fisher's exact tests to compare dichotomous variables.
b) *Multivariate analysis*
   To adjust for unequal distribution of parameters at baseline we will perform multivariate regression models, linear models in case of an interval scaled outcome and logistic regression in case of a dichotomous outcome.
c) *Interim analysis*
   We plan no interim analysis. In the case that the independent study monitoring board advises to suspend or stop the study, an interim analysis will be performed.

7.4 PLANNED SIGNIFICANCE LEVEL

The significance level will be <5% ($\alpha<0.05$).

7.5 HANDLING OF MISSING DATA OR OF DATA IN THE CASE OF SUBJECTS HALTING THE TRIAL PREMATURELY

In the case subjects halting the study prematurely, all recorded data will be used as far as meaningful for the evaluation and answering of our study questions**.** In case of missing or lost data, completion or recovery of data is pursued and eventually healthy volunteers or patients are invited for an extra visit to repeat or make up for the missing data. If amount of missing data is corrupting a meaningful statistical analysis new healthy volunteers or/and patients will be recruited.

7.6 DEFINITION OF THE EVALUATION GROUPS

Healthy volunteers and patients with LUTS are enrolled according to the inclusion/exclusion criteria.

8 STUDY SPECIFIC PREVENTIVE MEASURES AND DUTIES:

8.1 PREVENTIVE MEASURES AND DUTIES:

For study part I and II:
No consumption of caffeine, nicotine, alcohol, hallucinogens or use of hair styling products prior to Visits 2-3 (Part I) or 2-4 (Part II).

8.2 FINAL EXAMINATION

A follow-up telephone or e-mail interview will be performed with all subjects/patients 2 days after each measurement or in case of premature study termination to assess general well-being and potential adverse events or symptoms (see also separate follow up form). In case of adverse events, subjects will be appointed to an extra visit for further evaluation and treatment if applicable.

9 DUTIES ON THE PART OF THE INVESTIGATOR

9.1 CONFIRMATION

This study is conducted in compliance with the current study protocol, according to the principles of the Declaration of Helsinki (www.wma.net/en/20activities/10ethics/10helsinki/index.html), according to the guidelines on Good Clinical Practices (www.bag.admin.ch/themen/medizin/00701/00702/00703/index.html?lang=de), and according to Research with Human Subjects published by the Swiss Academy of Medical Sciences (www.samw.ch/en/News/News.html).
The investigators are aware of the GCP Guidelines, the effective legal regulations and accept them.

9.2 REPORTING OF SEVERE ADVERSE REACTIONS AND CHANGES TO THE PROTOCOL

All serious adverse events will be reported to the ethic committee within 15 days after the investigator has first knowledge of the serious adverse reactions. Serious adverse events that result in death or are life threatening will be reported expedited. The expedited reporting will occur not later than 7 days after the responsible investigator has first knowledge of the adverse reaction. This is for a preliminary report with another 8 days for completion of the full report.
Any change to the protocol and final reports will be submitted to the according ethical commission (KEK Zürich) in a timely manner.

9.3 OFFICIAL STATEMENT REGARDING DAMAGE COVERAGE OR ADDITIONAL COSTS

Participation in this study will not cause any additional costs for the patients or healthy volunteers. The costs of the study measurements and the examination are all covered by a research fund.
Study related damages to patients or healthy subjects are covered by an insurance that fulfills the legal requirements in Switzerland (Axa Winterthur, insurance policy number 14.050.565).

10 ETHICAL CONSIDERATIONS

10.1 EVALUATION OF THE RISK-BENEFIT RATIO

Due to the low risk profile of this study, the benefit clearly outweighs the risk. Healthy subjects and especially patients can benefit from the advanced diagnostic measures that will be used in this study without being exposed to health hazards.
Previous studies showed no significant adverse events and only rare cases of self-limited dysuria. The used devices and techniques are safe and have been frequently and safely used for investigations in humans either in several previous studies or during daily clinical routine.
This study can significantly contribute to a better understanding of LUT neurophysiology in healthy and pathological conditions, which certainly will help to improve future LUT diagnostics and to make treatment evaluation and development more effective.
In detail, our study on LUT SEPs has great potential to improve such investigation and provide normative data to make it more accessible for the clinical diagnostic use. That in turn would be beneficial for patients with LUTS in regard to a better functional and neurophysiological LUT diagnostic and consequently treatment selection.

**Benefit**	No	possible	probably
Is there a diagnostic use for the experimental subjects in the current study?		**X**<br>all subjects/ patients	
Is there any therapeutic use for the experimental subjects in the current study?	**X**		

**Risk**	Yes	No
High health risk		**x**
moderate health risk		**x**
Low health risk	**x**	
None health risk		**x**

<u>Expenditure of time:</u>
Healthy subjects: 8 hours allotted among 3 visits
Patients: 8 hours allotted among 3 visits
Patients receiving LUTS treatment: 10 hours allotted among 4 visits

10.2 DESCRIPTION OF WHY TRIAL SUBJECTS REQUIRING PARTICULAR PROTECTION HAVE BEEN INCLUDED

Healthy subjects are included to gain normative data and to be able to correctly interpret pathological findings in patients in relation to healthy controls.

11 QUALITY CONTROL AND QUALITY ASSURANCE: DESCRIPTION OF MEASURES

11.1 GUARANTEEING DIRECT ACCESS TO THE ORIGINAL DATA, PERMISSION FOR AUDITS TO BE CARRIED OUT, AND INSPECTIONS BY THE AUTHORITIES AND ETHICS COMMISSIONS

Only the principle investigators and the employees (see 1.2) have direct access to all data regarding this study. If required, access to all data will be granted to authorized personal (audits, health authorities, and the Kantonale Ethikkommission Zürich).

11.2 HANDLING, ARCHIVING, AND DESTRUCTION OF DATA AND SAMPLES

Data entry, analysis, and archiving are handled strictly confidential. The data are anonymized using a serial subject number. Only the informed consent form allows the allocation of the subject number with the true subject identity. The informed consent form exists as hard copy only. All other hard copy data (e.g. CRF, ICIQ-FLUTS/MLUTS, IPSS, and IIEF/FSFI questionnaires, bladder diary, SwissGerman OAB, ICIQ-LUTSqol, Qualiveen, HADS, MoCA) and electronic data (e.g. SEP latencies and amplitudes) are only identifiably by the serial subject number. The informed consent forms will be separately stored from the other documents in a separate study folder to guarantee anonymity.
The other hard copy data will be stored in the according study folders in the neuro-urology research office at the Balgrist University Hospital.
The electronic data are saved on the wissnet-server of the Paralab at the Balgrist University Hospital. Following completion of the study, all data will be archived for 10 years. Thereafter, hard copy data will be shredded and electronic data deleted.

11.3 DESCRIPTION OF THE DATA TO BE ENTERED DIRECTLY INTO THE CASE REPORT FORMS

The CRF includes data on:

- Body weight, body height
- Current and past medical history, concomitant medication, neurological status
- Result summary of urine analysis, pregnancy test, neuro-urological examination, and urodynamics
- Current perception and pain thresholds of SEPs and CHEPS, subjective subject response on electrical or contact heat stimulation
- Adverse events during measurements

REFERENCES

1. Coyne, K. S., Wein, A. J., Tubaro, A., Sexton, C. C., Thompson, C. L., Kopp, Z. S., Aiyer, L. P.: The burden of lower urinary tract symptoms: evaluating the effect of LUTS on health-related quality of life, anxiety and depression: EpiLUTS. BJU Int, 103 Suppl 3: 4, 2009

2. Irwin, D. E., Milsom, I., Kopp, Z., Abrams, P., Cardozo, L.: Impact of overactive bladder symptoms on employment, social interactions and emotional well-being in six European

countries. BJU Int, 97: 96, 2006

3. Irwin, D. E., Kopp, Z. S., Agatep, B., Milsom, I., Abrams, P.: Worldwide prevalence estimates of lower urinary tract symptoms, overactive bladder, urinary incontinence and bladder outlet obstruction. BJU Int, 108: 1132, 2011

4. Klotz, T., Bruggenjurgen, B., Burkart, M., Resch, A.: The economic costs of overactive bladder in Germany. Eur Urol, 51: 1654, 2007

5. Ganz, M. L., Smalarz, A. M., Krupski, T. L., Anger, J. T., Hu, J. C., Wittrup-Jensen, K. U., Pashos, C. L.: Economic costs of overactive bladder in the United States. Urology, 75: 526, 2010

6. Yoshimura, N.: Lower urinary tract symptoms (LUTS) and bladder afferent activity. Neurourol Urodyn, 26: 908, 2007

7. Fowler, C. J.: Bladder afferents and their role in the overactive bladder. Urology, 59: 37, 2002

8. Heeringa, R., van Koeveringe, G. A., Winkens, B., van Kerrebroeck, P. E., de Wachter, S. G.: Degree of urge, perception of bladder fullness and bladder volume--how are they related? J Urol, 186: 1352, 2011

9. De Wachter, S., Van Meel, T. D., Wyndaele, J. J.: Can a faked cystometry deceive patients in their perception of filling sensations? A study on the reliability of spontaneously reported cystometric filling sensations in patients with non-neurogenic lower urinary tract dysfunction. Neurourol Urodyn, 2007

10. Erdem, E., Akbay, E., Doruk, E., Cayan, S., Acar, D., Ulusoy, E.: How reliable are bladder perceptions during cystometry? Neurourol Urodyn, 23: 306, 2004

11. Bellucci, C. H., Wollner, J., Gregorini, F., Birnbock, D., Kozomara, M., Mehnert, U., Kessler, T. M.: Neurogenic lower urinary tract dysfunction--do we need same session repeat urodynamic investigations? J Urol, 187: 1318, 2012

12. Payne, C., Blaivas, J., Brown, J., Hirsch, M., Kusek, J., Peters, T., Steers, W., Stothers, M. L., Van Kerrebroeck, P., Weber, A.: ICI Committee 25: Research Methodology. In: Incontinence, Volume 1: Basics & Evaluation. Edited by P. Abrams, L. Cardozo, S. Khoury et al. Paris: Health Publication Ltd., vol. 1, pp. 97 - 148, 2005

13. Griffiths, D., Kondo, A., Bauer, S., Diamant, N., Liao, L., Lose, G., Schäfer, W., Yoshimura, N., Palmtag, H.: ICI Committee 7: Dynamic Testing. In: Incontinence, Volume 1: Basics & Evaluation. Edited by P. Abrams, L. Cardozo, S. Khoury et al. Paris: Health Publication Ltd., vol. 1, pp. 585 - 673, 2005

14. Frankl-Hochwart, L. V., Zuckerkandl, O.: Die nervösen Erkrankungen der Blase. In: Spezielle Pathologie und Therapie. Edited by v. Nothnagel. Wien: Holder, vol. 19, 1899

15. Van Meel, T. D., Wyndaele, J. J.: Reproducibility of electrical sensory testing in lower urinary tract at weekly intervals in healthy volunteers and women with non-neurogenic detrusor overactivity. Urology, 79: 526, 2012

16. Ukimura, O., Ushijima, S., Honjo, H., Iwata, T., Suzuki, K., Hirahara, N., Okihara, K., Mizutani, Y., Kawauchi, A., Miki, T.: Neuroselective current perception threshold

evaluation of bladder mucosal sensory function. Eur Urol, 45: 70, 2004

17. Kiesswetter, H.: Mucosal sensory threshold of urinary bladder and urethra measured electrically. Urologia internationalis, 32: 437, 1977

18. Frimodt-Moller, C.: A new method for quantitative evaluation of bladder sensibility. Scand J Urol Nephrol Suppl, 6: Suppl 15:135, 1972

19. Wyndaele, J. J., Van Eetvelde, B., Callens, D.: Comparison in young healthy volunteers of 3 different parameters of constant current stimulation used to determine sensory thresholds in the lower urinary tract. J Urol, 156: 1415, 1996

20. Wyndaele, J. J.: Studies on sensory threshold of different parts of the lower urinary tract measured electrically. Eur Urol, 19: 121, 1991

21. De Laet, K., De Wachter, S., Wyndaele, J. J.: Current perception thresholds in the lower urinary tract: Sine- and square-wave currents studied in young healthy volunteers. Neurourol Urodyn, 24: 261, 2005

22. Kenton, K., Simmons, J., FitzGerald, M. P., Lowenstein, L., Brubaker, L.: Urethral and bladder current perception thresholds: normative data in women. J Urol, 178: 189, 2007

23. Kiesswetter, H., Wober, G., Salah, S.: [The management of urinary and fecal incontinence due to a neurinoma of the cauda equina with an electrode implanted in the pelvic muscles (Caldwell) (author's transl)]. Journal of neurology, 215: 203, 1977

24. Kenton, K., Lowenstein, L., Simmons, J., Brubaker, L.: Aging and overactive bladder may be associated with loss of urethral sensation in women. Neurourol Urodyn, 26: 981, 2007

25. Hugonnet, C. L., Danuser, H., Springer, J. P., Studer, U. E.: Decreased sensitivity in the membranous urethra after orthotopic ileal bladder substitute. J Urol, 161: 418, 1999

26. Kessler, T. M., Studer, U. E., Burkhard, F. C.: Increased proximal urethral sensory threshold after radical pelvic surgery in women. Neurourol Urodyn, 26: 208, 2007

27. Kenton, K., Lowenstein, L., Brubaker, L.: Tolterodine causes measurable restoration of urethral sensation in women with urge urinary incontinence. Neurourol Urodyn, 29: 555, 2010

28. Lee, S. R., Kim, H. J., Kim, A., Kim, J. H.: Overactive bladder is not only overactive but also hypersensitive. Urology, 75: 1053, 2010

29. Van Meel, T. D., De Wachter, S., Wyndaele, J. J.: The effect of intravesical oxybutynin on the ice water test and on electrical perception thresholds in patients with neurogenic detrusor overactivity. Neurourol Urodyn, 29: 391, 2010

30. Vijaya, G., Digesu, G. A., Derpapas, A., Hendricken, C., Fernando, R., Khullar, V.: Antimuscarinic effects on current perception threshold: a prospective placebo control study. Neurourol Urodyn, 31: 75, 2012

31. Hedlund, P., Streng, T., Lee, T., Andersson, K. E.: Effects of tolterodine on afferent neurotransmission in normal and resiniferatoxin treated conscious rats. J Urol, 178: 326, 2007

32. Yokoyama, O., Yusup, A., Miwa, Y., Oyama, N., Aoki, Y., Akino, H.: Effects of tolterodine on an overactive bladder depend on suppression of C-fiber bladder afferent activity in rats. J Urol, 174: 2032, 2005

33. Dmochowski, R., Abrams, P., Marschall-Kehrel, D., Wang, J. T., Guan, Z.: Efficacy and tolerability of tolterodine extended release in male and female patients with overactive bladder. Eur Urol, 51: 1054, 2007

34. De Wachter, S., Wyndaele, J. J.: Quest for standardisation of electrical sensory testing in the lower urinary tract: the influence of technique related factors on bladder electrical thresholds. Neurourol Urodyn, 22: 118, 2003

35. Koga, K., Furue, H., Rashid, M. H., Takaki, A., Katafuchi, T., Yoshimura, M.: Selective activation of primary afferent fibers evaluated by sine-wave electrical stimulation. Mol Pain, 1: 13, 2005

36. Yokoyama, T., Nozaki, K., Fujita, O., Nose, H., Inoue, M., Kumon, H.: Role of C afferent fibers and monitoring of intravesical resiniferatoxin therapy for patients with idiopathic detrusor overactivity. J Urol, 172: 596, 2004

37. Fowler, C. J., Griffiths, D., de Groat, W. C.: The neural control of micturition. Nat Rev Neurosci, 9: 453, 2008

38. De Wachter, S., Wyndaele, J. J.: Can the sensory threshold toward electrical stimulation be used to quantify the subjective perception of bladder filling? A study in young healthy volunteers. Urology, 57: 655, 2001

39. Chiappa, K. H., Ropper, A. H.: Evoked potentials in clinical medicine (first of two parts). The New England journal of medicine, 306: 1140, 1982

40. Ganzer, H., Madersbacher, H., Rumpl, E.: Cortical evoked potentials by stimulation of the vesicourethral junction: clinical value and neurophysiological considerations. J Urol, 146: 118, 1991

41. Hansen, M. V., Ertekin, C., Larsson, L. E.: Cerebral evoked potentials after stimulation of the posterior urethra in man. Electroencephalogr Clin Neurophysiol, 77: 52, 1990

42. Sarica, Y., Karacan, I., Thornby, J. I., Hirshkowitz, M.: Cerebral responses evoked by stimulation of vesico-urethral junction in man: methodological evaluation of monopolar stimulation. Electroencephalogr Clin Neurophysiol, 65: 130, 1986

43. Sarica, Y., Karatas, M., Bozdemir, H., Karacan, I.: Cerebral responses elicited by stimulation of the vesico-urethral junction (VUJ) in diabetics. Electroencephalogr Clin Neurophysiol, 100: 55, 1996

44. Badr, G., Carlsson, C. A., Fall, M., Friberg, S., Lindstrom, L., Ohlsson, B.: Cortical evoked
potentials following stimulation of the urinary bladder in man. Electroencephalogr Clin Neurophysiol, 54: 494, 1982

45. Deltenre, P. F., Thiry, A. J.: Urinary bladder cortical evoked potentials in man: suitable stimulation techniques. British journal of urology, 64: 381, 1989

46. Hansen, M. V., Ertekin, C., Larsson, L. E., Pedersen, K.: A neurophysiological study of

patients undergoing radical prostatectomy. Scand J Urol Nephrol Suppl, 23: 267, 1989

47. Mehnert, U., Boy, S., Widmer-Simitovic, S., Reitz, A., Schurch, B.: The facilitatory effect of duloxetine combined with pelvic floor muscle training on the excitability of urethral sphincter motor neurons. Int Urogynecol J Pelvic Floor Dysfunct, 20: 659, 2009

48. Reitz, A., Haferkamp, A., Kyburz, T., Knapp, P. A., Wefer, B., Schurch, B.: The effect of tamsulosin on the resting tone and the contractile behaviour of the female urethra: a functional urodynamic study in healthy women. Eur Urol, 46: 235, 2004

49. Reitz, A., Knapp, P. A., Frey, S., Schurch, B.: Functional magnetic stimulation of the spinal cord--a urodynamic study in healthy humans. Neurourol Urodyn, 23: 148, 2004

50. Reitz, A., Knapp, P. A., Muntener, M., Schurch, B.: Oral nitric oxide donors: a new pharmacological approach to detrusor-sphincter dyssynergia in spinal cord injured patients? Eur Urol, 45: 516, 2004

51. Schurch, B., Reilly, I., Reitz, A., Curt, A.: Electrophysiological recordings during the peripheral nerve evaluation (PNE) test in complete spinal cord injury patients. World journal of urology, 20: 319, 2003

52. Mehnert, U., Michels, L., Zempleni, M. Z., Schurch, B., Kollias, S.: The supraspinal neural correlate of bladder cold sensation--an fMRI study. Human brain mapping, 32: 835, 2011

53. Zempleni, M. Z., Michels, L., Mehnert, U., Schurch, B., Kollias, S.: Cortical substrate of bladder control in SCI and the effect of peripheral pudendal stimulation. NeuroImage, 49: 2983, 2010

54. Mehnert, U., Boy, S., Svensson, J., Michels, L., Reitz, A., Candia, V., Kleiser, R., Kollias, S., Schurch, B.: Brain activation in response to bladder filling and simultaneous stimulation of the dorsal clitoral nerve--an fMRI study in healthy women. NeuroImage, 41: 682, 2008

55. Zhang, H., Reitz, A., Kollias, S., Summers, P., Curt, A., Schurch, B.: An fMRI study of the role of suprapontine brain structures in the voluntary voiding control induced by pelvic floor contraction. NeuroImage, 24: 174, 2005

56. Michels, L., Wöllner, J., Gregorini, F., Kurz, M., Schurch, B., Kessler, T. M., Kollias, S., Mehnert, U.: Supraspinal control of urine storage and micturition in men – an fMRI study. Annals of Neurology, under review, 2012

57. Reitz, A., Schmid, D. M., Curt, A., Knapp, P. A., Schurch, B.: Afferent fibers of the pudendal nerve modulate sympathetic neurons controlling the bladder neck. Neurourol Urodyn, 22: 597, 2003

58. Mehnert, U., Knapp, P. A., Mueller, N., Reitz, A., Schurch, B.: Heart rate variability: an objective measure of autonomic activity and bladder sensations during urodynamics. Neurourol Urodyn, 28: 313, 2009

59. Reitz, A., Schmid, D. M., Curt, A., Knapp, P. A., Schurch, B.: Sympathetic sudomotor skin
activity in human after complete spinal cord injury. Autonomic neuroscience : basic & clinical, 102: 78, 2002

60. Reitz, A., Curt, A., Schurch, B.: Organisation of the sympathetic skin response in spinal cord injury. Journal of neurology, neurosurgery, and psychiatry, 74: 1008, 2003

61. Schmid, D. M., Reitz, A., Curt, A., Hauri, D., Schurch, B.: Urethral evoked sympathetic skin responses and viscerosensory evoked potentials as diagnostic tools to evaluate urogenital autonomic afferent innervation in spinal cord injured patients. J Urol, 171: 1156, 2004

62. Schurch, B., Curt, A., Rossier, A. B.: The value of sympathetic skin response recordings in the assessment of the vesicourethral autonomic nervous dysfunction in spinal cord injured patients. J Urol, 157: 2230, 1997

63. Mehnert, U., Reitz, A., Youssef, S. A., Schurch, B.: Proof of principle: The effect of antimuscarinics on bladder filling sensations in healthy subjects--a placebo controlled double blind investigation using 4 and 8 mg tolterodine extended release. Neurourol Urodyn, 29: 464, 2010

64. Mehnert, U., Reitz, A., Ziegler, M., Knapp, P. A., Schurch, B.: Does tolterodine extended release affect the bladder electrical perception threshold? A placebo controlled, double-blind study with 4 and 8 mg in healthy volunteers. J Urol, 178: 2495, 2007

65. Boy, S., Schurch, B., Mehnert, U., Mehring, G., Karsenty, G., Reitz, A.: The effects of tolterodine on bladder-filling sensations and perception thresholds to intravesical electrical stimulation: method and initial results. BJU Int, 100: 574, 2007

66. Reitz, A., Schmid, D. M., Curt, A., Knapp, P. A., Jensen, K., Schurch, B.: Electrophysiological assessment of sensations arising from the bladder: are there objective criteria for subjective perceptions? J Urol, 169: 190, 2003

67. Gregorini, F., Wöllner, J., Schubert, M., Curt, A., Kessler, T. M., Mehnert, U.: Sensory evoked potentials of the human lower urinary tract. The Journal of Urology,, 2012

68. Gregorini, F., Wöllner, J., Schubert, M., Curt, A., Kessler, T. M., Mehnert, U.: Age-related differences in evoked potentials of the lower urinary tract. Neurourol Urodyn, under review, 2012

69. Tanosaki, M., Ozaki, I., Shimamura, H., Baba, M., Matsunaga, M.: Effects of aging on central conduction in somatosensory evoked potentials: evaluation of onset versus peak methods. Clinical neurophysiology : official journal of the International Federation of Clinical Neurophysiology, 110: 2094, 1999

70. Zumsteg, D., Wieser, H. G.: Effects of aging and sex on middle-latency somatosensory evoked potentials: normative data. Clinical neurophysiology : official journal of the International Federation of Clinical Neurophysiology, 113: 681, 2002

71. Gobbele, R., Dieckhofer, A., Thyerlei, D., Buchner, H., Waberski, T. D.: The impact of stimulus properties on low- and high-frequency median nerve somatosensory evoked potentials. Journal of clinical neurophysiology : official publication of the American Electroencephalographic Society, 25: 194, 2008

72. Sohn, S. Y., Seo, J. H., Min, Y., Seo, M. H., Eun, J. P., Song, K. J.: Changes in Dermatomal Somatosensory Evoked Potentials according to Stimulation Intensity and Severity of Carpal Tunnel Syndrome. Journal of Korean Neurosurgical Society, 51: 286, 2012

73. Gregorini, F., Wöllner, J., Schubert, M., Curt, A., Kessler, T. M., Mehnert, U.: Tibial nerve, pudendal nerve and sacral dermatome evoked potentials after electrical and contact heat stimulation in healthy subjects. PloS one, under review, 2012

74. Klem, G. H., Luders, H. O., Jasper, H. H., Elger, C.: The ten-twenty electrode system of the International Federation. The International Federation of Clinical Neurophysiology. Electroencephalography and clinical neurophysiology. Supplement, 52: 3, 1999

75. Pascual-Marqui, R. D., Esslen, M., Kochi, K., Lehmann, D.: Functional imaging with low-resolution brain electromagnetic tomography (LORETA): a review. Methods and findings in experimental and clinical pharmacology, 24 Suppl C: 91, 2002

76. Pascual-Marqui, R. D.: Standardized low-resolution brain electromagnetic tomography (sLORETA): technical details. Methods and findings in experimental and clinical pharmacology, 24 Suppl D: 5, 2002

77. Grave de Peralta Menendez, R., Gonzalez Andino, S. L., Morand, S., Michel, C. M., Landis, T.: Imaging the electrical activity of the brain: ELECTRA. Human brain mapping, 9: 1, 2000

78. Kiss G., Madersbacher H., Poewe W.: Cortical evoked potentials of the vesicourethraljunction-a predictor for the outcome of intravesical electrostimulation in patients with sensory and motor detrusor dysfunction: World Journal of Urology 16:308-312, 1998
