# Supplementary figures and images for: Does electrical stimulation in the lower urinary tract increase urine production? A randomised comparative proof-of-concept study in healthy volunteers
Source: PLoS One. 2019 May 24;14(5):e0217503. doi: 10.1371/journal.pone.0217503 (PMC6534346; doi:10.1371/journal.pone.0217503)

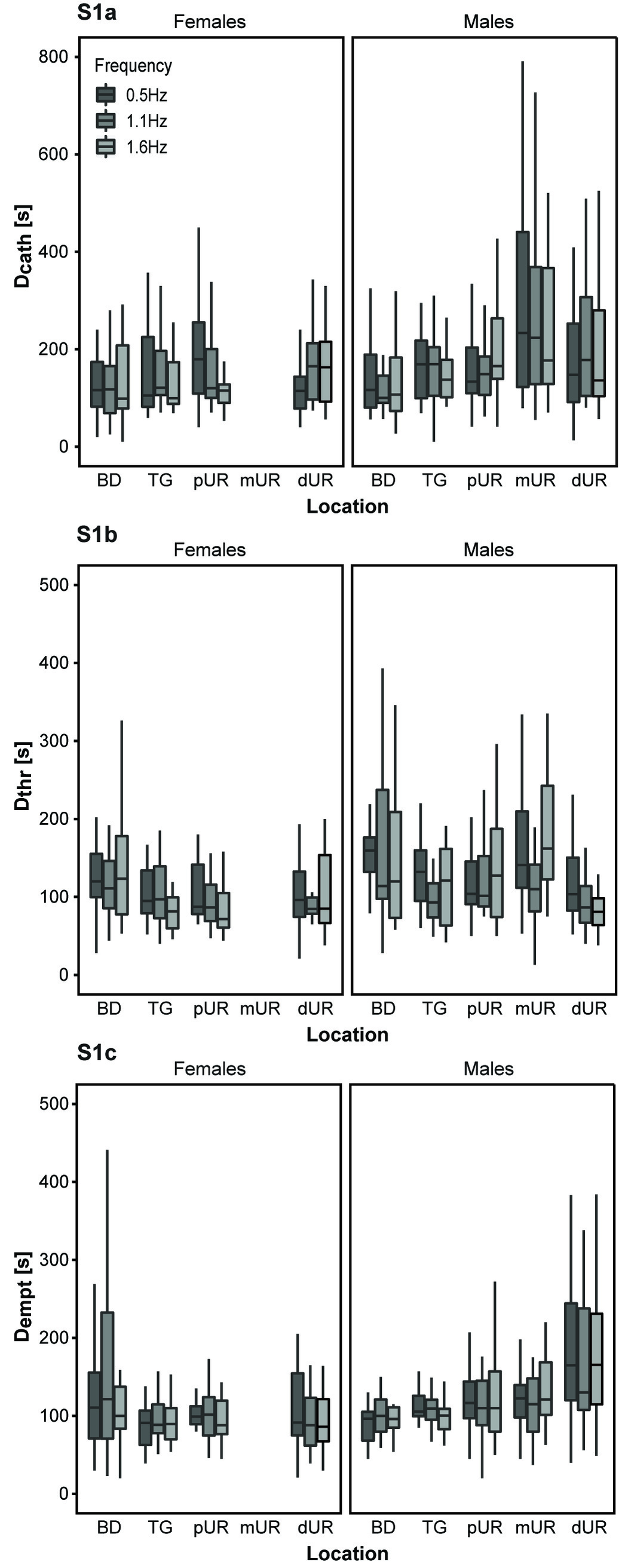

Supplement: S1 Fig — Dcath [s] (S1a) Dthr [s] (S1b), and Dempt [s] (S1c) for the three stimulation frequencies and five stimulation sites, stratified for gender. Outliers are not displayed. Dcath = time [s] used for catheter positioning at specific stimulation location; Dthr = time [s] used for current perception threshold (CPT) / pain threshold assessment and definition of absolute stimulation intensity (STIMINT); Dempt = time [s] used for bladder emptying. (TIF) [file pone.0217503.s003.tif]
