# Supplementary material for: Does electrical stimulation in the lower urinary tract increase urine production? A randomised comparative proof-of-concept study in healthy volunteers
Source: PLoS One. 2019 May 24;14(5):e0217503. doi: 10.1371/journal.pone.0217503 (PMC6534346; doi:10.1371/journal.pone.0217503)
Supplement: S1 Table — Locations: Bladder dome (BD, S1a), trigone (TG, S1b), proximal urethra (pUR, S1c), membranous urethra (mUR, S1d), distal urethra (dUR, S1e). Data are represented as (a) mean±standard deviation (SD), (b) median (range: minimum-maximum) or number of subjects (n) if appropriate. All subjects fulfilled predefined cut-offs for study inclusion: MoCA score ≥26, HADS ≤7 each, IPSS ≤7, BLD: 24hurinaryfrequencydrinkingvolume[mL]≤0.0045 with a maximum of 1x nocturia, mean volume per void >150mL and absence of urinary incontinence or urgency. (°) indicates significant gender differences p<0.05. + due to different scoring systems, female and male subjects have not been compared. ICIQ = International Consultation on Incontinence Modular Questionnaire, FLUTS = Female lower urinary tract symptoms, MLUTS = Male lower urinary tract symptoms, IPSS = International Prostate Symptom Score, OAB-q SF = The Overactive Bladder Questionnaire short-form, QoL = Quality of life, HADS = Hospital Anxiety and Depression Scale, MoCA = Montreal Cognitive Assessment. (DOCX) [file pone.0217503.s004.docx]

| **S1a) Baseline characteristics - BD** | **Women** | **Men** | **All** |
| --- | --- | --- | --- |
|  | **(n=10)** | **(n=10)** | **(n=20)** |
| ***Age [years]*** ^b^ | 22.7 (18.8 - 26.9) | 22.1 (19.1 - 31.9) | 22.5 (18.8 - 31.9) |
| ***Height [m]*** ^b^ | 1.7 (1.6 - 1.8) | 1.8 (1.7-1.9) | 1.7 (1.6 - 1.9)° |
| ***Weight [kg]*** ^b^ | 65.5 (59 - 72) | 73 (58 - 95) | 68.5 (58 - 95)° |
| ***3-day bladder diary*** |  |  |  |
| Micturition frequency per 24 hours ^a^ | 6.4±2.3 | 5.0±1.6 | 5.7±2.1 |
| Micturition volume per micturition [mL] ^b^ | 312 (238 - 550) | 349 (246 - 517) | 324 (238 - 550) |
| Fluid intake per 24 hours [mL] ^b^ | 2140 (1373 - 4287) | 2048 (783 - 4183) | 2100 (783 - 4287) |
| ***Questionnaires*** |  |  |  |
| ICIQ-FLUTS/MLUTS^+^ |  |  |  |
| Filling symptoms ^b^ | 1.5 (0 - 4) |  |  |
| Voiding symptoms ^b^ | 0 (0 - 2) | 1 (0 - 4) |  |
| Incontinence symptoms ^b^ | 0 (0 - 2) | 1 (0 - 4) |  |
| IPSS ^b^ |  | 1 (0 - 3) |  |
| OAB-q SF |  |  |  |
| Symptoms ^b^ | 6 (6 - 7) | 6 (6 - 15) | 6 (6 - 15) |
| QoL ^b^ | 13 (13 - 14) | 13 (13 - 17) | 13 (13 - 17) |
| HADS |  |  |  |
| Anxiety ^b^ | 2.5 (0 - 7) | 3 (0 - 4) | 3 (0 - 7) |
| Depression ^b^ | 0 (0 - 4) | 2 (0 - 5) | 1 (0 - 5) |
| MoCA ^b^ | 29 (26 - 31) | 28.5 (26 - 30) | 29 (26 - 31) |
| ***Neuro-Urological examination*** |  |  |  |
| Urogenital sensation  (n intact/impaired) | 10/0 | 10/0 | 20/0 |
| Bulbocavernosus reflex (n intact/impaired) | 10/0 | 10/0 | 20/0 |
| Anal reflex  (n intact/impaired) | 10/0 | 10/0 | 20/0 |
| Anal sphincter tone  (n intact/impaired) | 10/0 | 10/0 | 20/0 |
| Anal squeeze response  (n intact/impaired) | 10/0 | 10/0 | 20/0 |
| ***Free uroflowmetry*** |  |  |  |
| Voided volume [mL] ^b^ | 360 (171 - 1243) | 350 (95 - 658) | 350 (95 - 1243) |
| Maximum flow rate [mL/s] ^b^ | 53.6 (27.4 - 62.9) | 33.9 (11.1 - 77.4) | 39.5 (11.1 - 77.4) |
| Post void residual [mL] ^b^ | 5.4 (0 - 38.1) | 2.2 (0 - 34) | 2.2 (0 - 38.1) |

| **S1b) Baseline characteristics - TG** | **Women** | **Men** | **All** |
| --- | --- | --- | --- |
|  | **(n=10)** | **(n=10)** | **(n=20)** |
| ***Age [years]*** ^b^ | 23.7 (18.3 - 35.8) | 25.2 (18.3 - 32.2) | 24.3 (18.3 - 35.8) |
| ***Height [m]*** ^b^ | 1.7 (1.6 - 1.9) | 1.7 (1.6 - 2.0) | 1.7 (1.6 - 2.0) |
| ***Weight [kg]*** ^b^ | 63 (48 - 85) | 66 (59 - 98) | 65.5 (48 - 98) |
| ***3-day bladder diary*** |  |  |  |
| Micturition frequency per 24 hours ^a^ | 6.1±1.7 | 5.9±2.8 | 6±2.2 |
| Micturition volume per micturition [mL] ^b^ | 376 (178 - 487) | 303 (209 - 1057) | 338 (178 - 1057) |
| Fluid intake per 24 hours [mL] ^b^ | 2192 (1067 - 3283) | 1887 (1567 - 7953) | 2000 (1067 - 7953) |
| ***Questionnaires*** |  |  |  |
| ICIQ-FLUTS/MLUTS^+^ |  |  |  |
| Filling symptoms ^b^ | 1 (0 - 3) |  |  |
| Voiding symptoms ^b^ | 0 (0 - 3) | 2 (0 - 6) |  |
| Incontinence symptoms ^b^ | 0 (0 - 2) | 1 (0 - 3) |  |
| IPSS ^b^ |  | 1.5 (0 - 6) |  |
| OAB-q SF |  |  |  |
| Symptoms ^b^ | 8 (6 - 10) | 6 (6 - 16) | 6.5 (6 - 16) |
| QoL ^b^ | 13.5 (13 - 17) | 13 (13 - 18) | 13 (13 - 18) |
| HADS |  |  |  |
| Anxiety ^b^ | 4 (1 - 6) | 3 (0 - 7) | 3.5 (0 - 7) |
| Depression ^b^ | 1.5 (0 - 6) | 0.5 (0 - 4) | 1 (0 - 6) |
| MoCA ^b^ | 28 (27 - 30) | 28.5 (27 - 30) | 28 (27 - 30) |
| ***Neuro-Urological examination*** |  |  |  |
| Urogenital sensation  (n intact/impaired) | 10/0 | 10/0 | 20/0 |
| Bulbocavernosus reflex (n intact/impaired) | 10/0 | 9/1 | 19/1 |
| Anal reflex  (n intact/impaired) | 10/0 | 10/0 | 20/0 |
| Anal sphincter tone  (n intact/impaired) | 10/0 | 10/0 | 20/0 |
| Anal squeeze response  (n intact/impaired) | 10/0 | 10/0 | 20/0 |
| ***Free uroflowmetry*** |  |  |  |
| Voided volume [mL] ^b^ | 465 (207 - 1195) | 429 (170 - 1195) | 462 (170 - 1195) |
| Maximum flow rate [mL/s] ^b^ | 42.7 (14.5 - 79.4) | 31.1 (22.3 - 34.3) | 33.0 (14.5 - 79.4)° |
| Post void residual [mL] ^b^ | 1.5 (0 - 64.5) | 2.0 (0 - 102.7) | 2.0 (0 - 102.7) |

| **S1c) Baseline characteristics - pUR** | **Women** | **Men** | **All** |
| --- | --- | --- | --- |
|  | **(n=10)** | **(n=10)** | **(n=20)** |
| ***Age [years]*** ^b^ | 22.7 (18.5 - 27.0) | 23.4 (19.6 - 33.2) | 22.9 (18.5 - 33.2) |
| ***Height [m]*** ^b^ | 1.7 (1.6 - 1.8) | 1.8 (1.7 - 1.9) | 1.7 (1.6 - 1.9)° |
| ***Weight [kg]*** ^b^ | 58.5 (51 - 80) | 76.5 (57 - 126) | 66 (51 - 126)° |
| ***3-day bladder diary*** |  |  |  |
| Micturition frequency per 24 hours ^a^ | 6.3±1.1 | 4.8±1.7 | 5.6±1.6° |
| Micturition volume per micturition [mL] ^b^ | 232 (162 - 399) | 343 (246 - 517) | 301 (162 - 517) |
| Fluid intake per 24 hours [mL] ^b^ | 1828 (1050 - 3427) | 2265 (783 - 3333) | 1995 (783 - 3427) |
| ***Questionnaires*** |  |  |  |
| ICIQ-FLUTS/MLUTS^+^ |  |  |  |
| Filling symptoms ^b^ | 1 (0 - 3) |  |  |
| Voiding symptoms ^b^ | 1 (0 - 3) | 0 (0 - 5) |  |
| Incontinence symptoms ^b^ | 0 (0 - 0) | 0 (0 - 3) |  |
| IPSS ^b^ |  | 1.5 (0 - 5) |  |
| OAB-q SF |  |  |  |
| Symptoms ^b^ | 6.5 (6 - 10) | 6 (6 - 6) | 6 (6 - 10)° |
| QoL ^b^ | 13 (13 - 17) | 13 (13 - 15) | 13 (13 - 17) |
| HADS |  |  |  |
| Anxiety ^b^ | 4 (1 - 6) | 3.5 (0 - 7) | 4 (0 - 7) |
| Depression ^b^ | 1.5 (0 - 4) | 2.5 (0-6) | 2 (0-6) |
| MoCA ^b^ | 28.5 (28 - 30) | 29 (26 - 30) | 29 (26 - 30) |
| ***Neuro-Urological examination*** |  |  |  |
| Urogenital sensation  (n intact/impaired) | 10/0 | 10/0 | 20/0 |
| Bulbocavernosus reflex (n intact/impaired) | 10/0 | 10/0 | 20/0 |
| Anal reflex  (n intact/impaired) | 10/0 | 10/0 | 20/0 |
| Anal sphincter tone  (n intact/impaired) | 10/0 | 10/0 | 20/0 |
| Anal squeeze response  (n intact/impaired) | 10/0 | 10/0 | 20/0 |
| ***Free uroflowmetry*** |  |  |  |
| Voided volume [mL] ^b^ | 400 (161 - 896) | 477 (332 - 988) | 441 (161- 988) |
| Maximum flow rate [mL/s] ^b^ | 33.4 (12.4 - 52.6) | 37.3 (19.7 - 58.1) | 37.1 (12.4 - 58.1) |
| Post void residual [mL] ^b^ | 1.5 (0 - 6.3) | 17.8 (0 - 117) | 4.5 (0 - 117) |

| **S1d) Baseline characteristics - mUR** | **Women** | **Men** | **All** |
| --- | --- | --- | --- |
|  | **(n=0)** | **(n=10)** | **(n=10)** |
| ***Age [years]*** ^b^ |  | 24.8 (21.4 - 28.1) |  |
| ***Height [m]*** ^b^ |  | 1.8 (1.6 - 2.0) |  |
| ***Weight [kg]*** ^b^ |  | 75 (66 - 87) |  |
| ***3-day bladder diary*** |  |  |  |
| Micturition frequency per 24 hours ^a^ |  | 5.4±1.5 |  |
| Micturition volume per micturition [mL] ^b^ |  | 326 (262 - 600) |  |
| Fluid intake per 24 hours [mL] ^b^ |  | 2207 (1753 - 4183) |  |
| ***Questionnaires*** |  |  |  |
| ICIQ-FLUTS/MLUTS^+^ |  |  |  |
| Filling symptoms ^b^ |  |  | . |
| Voiding symptoms ^b^ |  | 1.5 (0 - 4) | . |
| Incontinence symptoms ^b^ |  | 0.5 (0 - 3) | . |
| IPSS ^b^ | . | 2.5 (0 - 4) | . |
| OAB-q SF |  |  |  |
| Symptoms ^b^ |  | 6 (6 - 11) |  |
| QoL ^b^ |  | 13 (13 - 16) |  |
| HADS |  |  |  |
| Anxiety ^b^ |  | 2.5 (1 - 7) |  |
| Depression ^b^ |  | 0 (0 - 6) |  |
| MoCA ^b^ |  | 28 (26 - 30) |  |
| ***Neuro-Urological examination*** |  |  |  |
| Urogenital sensation  (n intact/impaired) |  | 10/0 |  |
| Bulbocavernosus reflex (n intact/impaired) |  | 10/0 |  |
| Anal reflex  (n intact/impaired) |  | 10/0 |  |
| Anal sphincter tone  (n intact/impaired) |  | 10/0 |  |
| Anal squeeze response  (n intact/impaired) |  | 10/0 |  |
| ***Free uroflowmetry*** |  |  |  |
| Voided volume [mL] ^b^ |  | 352 (217 - 857) |  |
| Maximum flow rate [mL/s] ^b^ |  | 27.4 (17.2 - 65) |  |
| Post void residual [mL] ^b^ |  | 8.3 (0 - 31.5) |  |

| **S1e) Baseline characteristics - dUR** | **Women** | **Men** | **All** |
| --- | --- | --- | --- |
|  | **(n=10)** | **(n=10)** | **(n=20)** |
| ***Age [years]*** ^b^ | 25.4 (18.3 - 29.2) | 22.6 (19.5 - 34.1) | 23.5 (18.3 - 34.1) |
| ***Height [m]*** ^b^ | 1.7 (1.6 - 1.8) | 1.8 (1.7 - 1.9) | 1.7 (1.6 - 1.9)° |
| ***Weight [kg]*** ^b^ | 57 (48 - 80) | 76 (65 - 126) | 66.5 (48 - 126)° |
| ***3-day bladder diary*** ^b^ |  |  |  |
| Micturition frequency per 24 hours ^a^ | 7.0±1.5 | 4.9±1.7 | 6.0±1.9° |
| Micturition volume per micturition [mL] ^b^ | 304 (254 - 717) | 394 (224 - 538) | 343 (224 - 717) |
| Fluid intake per 24 hours [mL] ^b^ | 2466 (1483 - 5717) | 2450 (1100 - 2985) | 2450 (1100 - 5717) |
| ***Questionnaires*** |  |  |  |
| ICIQ-FLUTS/MLUTS^+^ |  |  |  |
| Filling symptoms ^b^ | 1 (0 - 5) |  |  |
| Voiding symptoms ^b^ | 0.5 (0 - 3) | 1.5 (0 - 5) |  |
| Incontinence symptoms ^b^ | 0 (0 - 1) | 0 (0 - 2) |  |
| IPSS ^b^ |  | 1 (0 - 3) |  |
| OAB-q SF |  |  |  |
| Symptoms ^b^ | 6.5 (6 - 11) | 6 (6 - 7) | 6 (6 - 11) |
| QoL ^b^ | 13 (13 - 15) | 13 (13 - 14) | 13 (13 - 15) |
| HADS |  |  |  |
| Anxiety ^b^ | 3.5 (1 - 7) | 1 (0-5) | 2.5 (0 - 7) |
| Depression ^b^ | 1 (0 - 3) | 0 (0-3) | 0.5 (0 - 3) |
| MoCA ^b^ | 29 (27 - 30) | 29 (26-30) | 29 (26 - 30) |
| ***Neuro-Urological examination*** |  |  |  |
| Urogenital sensation  (n intact/impaired) | 10/0 | 10/0 | 20/0 |
| Bulbocavernosus reflex (n intact/impaired) | 10/0 | 10/0 | 20/0 |
| Anal reflex  (n intact/impaired) | 10/0 | 10/0 | 20/0 |
| Anal sphincter tone  (n intact/impaired) | 10/0 | 10/0 | 20/0 |
| Anal squeeze response  (n intact/impaired) | 10/0 | 10/0 | 20/0 |
| ***Free uroflowmetry*** |  |  |  |
| Voided volume [mL] ^b^ | 494 (226 - 1195) | 489 (174 - 997) | 489 (174 - 1195) |
| Maximum flow rate [mL/s] ^b^ | 39.3 (16.9 - 72.4) | 26.6 (20.3 - 63.5) | 35.7 (16.9 - 72.4) |
| Post void residual [mL] ^b^ | 1.5 (0 - 38.1) | 1.8 (0 - 117) | 1.8 (0 - 117) |
