# Supplementary material for: Does electrical stimulation in the lower urinary tract increase urine production? A randomised comparative proof-of-concept study in healthy volunteers
Source: PLoS One. 2019 May 24;14(5):e0217503. doi: 10.1371/journal.pone.0217503 (PMC6534346; doi:10.1371/journal.pone.0217503)
Supplement: S2 Table — DF: degrees of freedom; n: number of subjects; SD: standard deviation; SE: standard error; Simulated LRT: simulated likelihood ratio test; aBaseline = 0Hz; bBaseline = 0mA; cBaseline = Trigone; dBaseline = first stimulation; eBaseline = 0 years; fBaseline = females; gBaseline = Visit 1; Asterisk (*) indicates statistical significance p<0.05. (DOCX) [file pone.0217503.s005.docx]

| Name | |  | | Estimate | SE | t-value | DF | p-value |  | Confidence interval (95%) | | |  | | Simulated LRT |
| --- | --- | --- | --- | --- | --- | --- | --- | --- | --- | --- | --- | --- | --- | --- | --- |
|  | |  | |  |  |  |  |  |  |  |  |  |  | |  |
|  | |  | |  |  |  |  |  |  | Lower | Upper | |  | | p-value |
| **Fixed effects** | |  | |  |  |  |  |  |  |  |  | |  | |  |
| (Intercept) | |  | | 4.336 | 2.954 | 1.468 | 510 | 0.143 |  | -1.467 | 10.139 | |  | |  |
| Stimulation frequency^a^ | |  | | 3.408 | 0.433 | 7.862 | 510 | <0.001 |  | 2.556 | 4.259 | |  | | <0.001* |
| Stimulation intensity^b^ | |  | | 0.037 | 0.030 | 1.233 | 510 | 0.218 |  | -0.022 | 0.095 | |  | | 0.240 |
| Location^c^ | |  | |  |  |  |  |  |  |  |  | |  | | 0.147 |
|  | *bladder dome* | | | -0.832 | 1.206 | -0.690 | 510 | 0.490 |  | -3.202 | 1.537 | |  | |  |
|  | *proximal urethra* | | | 0.309 | 1.193 | 0.259 | 510 | 0.796 |  | -2.034 | 2.652 | |  | |  |
|  | *membranous urethra* | | | -0.218 | 1.520 | -0.143 | 510 | 0.886 |  | -3.203 | 2.767 | |  | |  |
|  | *distal urethra* | | | -2.682 | 1.190 | -2.254 | 510 | 0.025 |  | -5.019 | -0.344 | |  | |  |
| Stimulation order^d^ | |  | |  |  |  |  |  |  |  |  | |  | | 0.033* |
|  | *2nd stimulation* | | | -0.949 | 0.464 | -2.044 | 510 | 0.041 |  | -1.861 | -0.037 | |  | |  |
|  | *3rd stimulation* | | | -1.183 | 0.473 | -2.500 | 510 | 0.013 |  | -2.113 | -0.253 | |  | |  |
| Age^e^ | |  | | 0.076 | 0.111 | 0.685 | 510 | 0.493 |  | -0.141 | 0.293 | |  | | 0.501 |
| Gender^f^ | |  | | -2.126 | 0.864 | -2.461 | 510 | 0.014 |  | -3.823 | -0.428 | |  | | 0.019* |
| Visit^g^ | |  | | 0.239 | 0.388 | 0.616 | 510 | 0.538 |  | -0.524 | 1.002 | |  | | 0.550 |
| **Random effects** | |  | |  |  |  |  |  |  |  |  | |  | |  |
| Group | |  | | Name | SD |  |  |  |  |  |  | |  | |  |
| Subject | |  | | (Intercept) | 3.293 |  |  |  |  |  |  | |  | |  |
| Residual | |  | |  | 4.289 |  |  |  |  |  |  | |  | |  |
| n | 90 | | |  |  |  |  |  |  |  |  | |  | |  |
| Adjusted R^2^ | 0.417 | | |  |  |  |  |  |  |  |  | |  | |  |
|  | | | | | | | | |  |  |  |  | |  |  |
|  | | | | | | | | | | | | | | | |
|  |  |  |  |  |  |  |  |  |  |  |  |  |  |  |  |
|  | | |  |  |  |  |  |  |  |  |  | |  | |  |
