# Supplementary material for: Does electrical stimulation in the lower urinary tract increase urine production? A randomised comparative proof-of-concept study in healthy volunteers
Source: PLoS One. 2019 May 24;14(5):e0217503. doi: 10.1371/journal.pone.0217503 (PMC6534346; doi:10.1371/journal.pone.0217503)
Supplement: S3 Table — DF: degrees of freedom; n: number of subjects; SD: standard deviation; SE: standard error; Simulated LRT: simulated likelihood ratio test; aBaseline = 0Hz; bBaseline = 0mA; cBaseline = Trigone; dBaseline = first stimulation; eBaseline = 0 years; fBaseline = females; gBaseline = Visit 1; Asterisk (*) indicates statistical significance p<0.05. (DOCX) [file pone.0217503.s006.docx]

| Name | |  | Estimate | SE | t-value | DF | p-value |  | Confidence interval (95%) | |  | Simulated LRT |
| --- | --- | --- | --- | --- | --- | --- | --- | --- | --- | --- | --- | --- |
|  | |  |  |  |  |  |  |  |  |  |  |  |
|  | |  |  |  |  |  |  |  | Lower | Upper |  | p-value |
| **Fixed effects** | |  |  |  |  |  |  |  |  |  |  |  |
| (Intercept) | |  | 3.529 | 4.340 | 0.813 | 468 | 0.417 |  | -4.999 | 12.057 |  |  |
| Stimulation frequency^a^ | |  | 4.637 | 0.608 | 7.633 | 468 | <0.001 |  | 3.443 | 5.831 |  | <0.001* |
| Stimulation intensity^b^ | |  | 0.119 | 0.045 | 2.647 | 468 | 0.008 |  | 0.031 | 0.208 |  | 0.012* |
| Location^c^ | |  |  |  |  |  |  |  |  |  |  | 0.170 |
|  | *bladder dome* | | -1.759 | 1.740 | -1.011 | 468 | 0.313 |  | -5.178 | 1.660 |  |  |
|  | *proximal urethra* | | 0.207 | 1.715 | 0.120 | 468 | 0.904 |  | -3.164 | 3.577 |  |  |
|  | *distal urethra* | | -3.326 | 1.719 | -1.935 | 468 | 0.054 |  | -6.703 | 0.051 |  |  |
| Stimulation order^d^ | |  |  |  |  |  |  |  |  |  |  | 0.008* |
|  | *2nd stimulation* | | -1.482 | 0.645 | -2.297 | 468 | 0.022 |  | -2.751 | -0.214 |  |  |
|  | *3rd stimulation* | | -1.972 | 0.657 | -3.002 | 468 | 0.003 |  | -3.262 | -0.681 |  |  |
| Age^e^ | |  | 0.073 | 0.164 | 0.447 | 468 | 0.655 |  | -0.248 | 0.394 |  | 0.665 |
| Gender^f^ | |  | -2.703 | 1.248 | -2.166 | 468 | 0.031 |  | -5.155 | -0.251 |  | 0.043* |
| Visit^g^ | |  | 0.368 | 0.542 | 0.678 | 468 | 0.498 |  | -0.698 | 1.434 |  | 0.499 |
| **Random effects** | |  |  |  |  |  |  |  |  |  |  |  |
| Group | |  | Name | SD |  |  |  |  |  |  |  |  |
| Subject | |  | (Intercept) | 4.865 |  |  |  |  |  |  |  |  |
| Residual | |  |  | 5.746 |  |  |  |  |  |  |  |  |
| n | 80 | |  |  |  |  |  |  |  |  |  |  |
| Adjusted R^2^ | 0.466 | |  |  |  |  |  |  |  |  |  |  |
