# Supplementary material for: Does electrical stimulation in the lower urinary tract increase urine production? A randomised comparative proof-of-concept study in healthy volunteers
Source: PLoS One. 2019 May 24;14(5):e0217503. doi: 10.1371/journal.pone.0217503 (PMC6534346; doi:10.1371/journal.pone.0217503)
Supplement: S4 Table — DF: degrees of freedom; n: number of subjects; SD: standard deviation; SE: standard error; Simulated LRT: simulated likelihood ratio test; aBaseline = 0Hz; bBaseline = 0mA; cBaseline = Trigone; dBaseline = first stimulation; eBaseline = 0 years; fBaseline = females; gBaseline = Visit 1; Asterisk (*) indicates statistical significance p<0.05. (DOCX) [file pone.0217503.s007.docx]

| Name | |  | Estimate | SE | t-value | DF | p-value |  | Confidence interval (95%) | |  | Simulated LRT |
| --- | --- | --- | --- | --- | --- | --- | --- | --- | --- | --- | --- | --- |
|  | |  |  |  |  |  |  |  |  |  |  |  |
|  | |  |  |  |  |  |  |  | Lower | Upper |  | p-value |
| **Fixed effects** | |  |  |  |  |  |  |  |  |  |  |  |
| (Intercept) | |  | 171.792 | 36.879 | 4.658 | 527 | <0.001 |  | 99.344 | 244.240 |  |  |
| Stimulation frequency^a^ | |  | -47.650 | 5.521 | -8.630 | 527 | <0.001 |  | -58.496 | -36.804 |  | <0.001* |
| Stimulation intensity^b^ | |  | 0.726 | 0.378 | 1.920 | 527 | 0.055 |  | -0.017 | 1.470 |  | 0.068 |
| Location^c^ | |  |  |  |  |  |  |  |  |  |  | 0.474 |
|  | *bladder dome* | | -17.253 | 14.969 | -1.153 | 527 | 0.250 |  | -46.658 | 12.153 |  |  |
|  | *proximal urethra* | | -19.376 | 14.777 | -1.311 | 527 | 0.190 |  | -48.404 | 9.652 |  |  |
|  | *membranous urethra* | | -7.014 | 18.881 | -0.372 | 527 | 0.710 |  | -44.105 | 30.077 |  |  |
|  | *distal urethra* | | -27.408 | 14.807 | -1.851 | 527 | 0.065 |  | -56.496 | 1.680 |  |  |
| Stimulation order^d^ | |  |  |  |  |  |  |  |  |  |  | <0.001* |
|  | *2nd stimulation* | | -24.260 | 5.934 | -4.088 | 527 | <0.001 |  | -35.917 | -12.602 |  |  |
|  | *3rd stimulation* | | -29.471 | 6.041 | -4.879 | 527 | <0.001 |  | -41.338 | -17.604 |  |  |
| Age^e^ | |  | 0.851 | 1.377 | 0.618 | 527 | 0.537 |  | -1.854 | 3.556 |  | 0.557 |
| Gender^f^ | |  | -30.755 | 10.744 | -2.862 | 527 | 0.004 |  | -51.863 | -9.648 |  | 0.006* |
| Visit^g^ | |  | 4.247 | 4.960 | 0.856 | 527 | 0.392 |  | -5.497 | 13.992 |  | 0.395 |
| **Random effects** | |  |  |  |  |  |  |  |  |  |  |  |
| Group | |  | Name | SD |  |  |  |  |  |  |  |  |
| Subject | |  | (Intercept) | 40.563 |  |  |  |  |  |  |  |  |
| Residual | |  |  | 55.916 |  |  |  |  |  |  |  |  |
| n | 90 | |  |  |  |  |  |  |  |  |  |  |
| Adjusted R^2^ | 0.432 | |  |  |  |  |  |  |  |  |  |  |
