# Supplementary material for: Does electrical stimulation in the lower urinary tract increase urine production? A randomised comparative proof-of-concept study in healthy volunteers
Source: PLoS One. 2019 May 24;14(5):e0217503. doi: 10.1371/journal.pone.0217503 (PMC6534346; doi:10.1371/journal.pone.0217503)
Supplement: S5 Table — DT1-baseline: all daytime urine volumes from bladder diary; DF: degrees of freedom; n: number of subjects; SD: standard deviation; SE: standard error; Simulated LRT: simulated likelihood ratio test; aBaseline = 0Hz; bBaseline = 0mA; cBaseline = Trigone; dBaseline = first stimulation; eBaseline = 0 years; fBaseline = females; gBaseline = Visit 1; Asterisk (*) indicates statistical significance p<0.05. (DOCX) [file pone.0217503.s008.docx]

| Name | |  | | Estimate | SE | t-value | DF | p-value |  | Confidence interval (95%) | |  | Simulated LRT |
| --- | --- | --- | --- | --- | --- | --- | --- | --- | --- | --- | --- | --- | --- |
|  | |  | |  |  |  |  |  |  |  |  |  |  |
|  | |  | |  |  |  |  |  |  | Lower | Upper |  | p-value |
| **Fixed effects** | |  | |  |  |  |  |  |  |  |  |  |  |
| (Intercept) | |  | | 0.962 | 2.587 | 0.372 | 527 | 0.710 |  | -4.119 | 6.044 |  |  |
| Stimulation frequency^a^ | |  | | 2.767 | 0.363 | 7.614 | 527 | <0.001 |  | 2.053 | 3.481 |  | <0.001* |
| Stimulation intensity^b^ | |  | | 0.055 | 0.026 | 2.138 | 527 | 0.033 |  | 0.004 | 0.105 |  | 0.040* |
| Location^c^ | |  | |  |  |  |  |  |  |  |  |  | 0.197 |
|  | *bladder dome* | | | -1.115 | 1.054 | -1.058 | 527 | 0.290 |  | -3.186 | 0.955 |  |  |
|  | *proximal urethra* | | | -0.072 | 1.041 | -0.069 | 527 | 0.945 |  | -2.118 | 1.973 |  |  |
|  | *membranous urethra* | | | -0.979 | 1.330 | -0.737 | 527 | 0.462 |  | -3.592 | 1.633 |  |  |
|  | *distal urethra* | | | -2.357 | 1.043 | -2.260 | 527 | 0.024 |  | -4.405 | -0.308 |  |  |
| Stimulation order^d^ | |  | |  |  |  |  |  |  |  |  |  | 0.006* |
|  | *2nd stimulation* | | | -0.957 | 0.390 | -2.453 | 527 | 0.014 |  | -1.723 | -0.191 |  |  |
|  | *3rd stimulation* | | | -1.190 | 0.397 | -2.994 | 527 | 0.003 |  | -1.971 | -0.409 |  |  |
| Age^e^ | |  | | 0.120 | 0.097 | 1.237 | 527 | 0.217 |  | -0.071 | 0.311 |  | 0.233 |
| Gender^f^ | |  | | -1.610 | 0.756 | -2.131 | 527 | 0.034 |  | -3.094 | -0.125 |  | 0.045* |
| Visit^g^ | |  | | 0.036 | 0.326 | 0.111 | 527 | 0.912 |  | -0.605 | 0.678 |  | 0.913 |
| **Random effects** | |  | |  |  |  |  |  |  |  |  |  |  |
| Group | |  | | Name | SD |  |  |  |  |  |  |  |  |
| Subject | |  | | (Intercept) | 2.917 |  |  |  |  |  |  |  |  |
| Residual | |  | |  | 3.675 |  |  |  |  |  |  |  |  |
| n | 90 | | |  |  |  |  |  |  |  |  |  |  |
| Adjusted R^2^ | 0.434 | | |  |  |  |  |  |  |  |  |  |  |
