# Supplementary material for: Does electrical stimulation in the lower urinary tract increase urine production? A randomised comparative proof-of-concept study in healthy volunteers
Source: PLoS One. 2019 May 24;14(5):e0217503. doi: 10.1371/journal.pone.0217503 (PMC6534346; doi:10.1371/journal.pone.0217503)
Supplement: S6 Table — DT2-baseline: daytime urine volumes from bladder diary (BLD) minus the first morning urine volume; DF: degrees of freedom; n: number of subjects; SD: standard deviation; SE: standard error; Simulated LRT: simulated likelihood ratio test; aBaseline = 0Hz; bBaseline = 0mA; cBaseline = Trigone; dBaseline = first stimulation; eBaseline = 0 years; fBaseline = females; gBaseline = Visit 1; Asterisk (*) indicates statistical significance p<0.05. (DOCX) [file pone.0217503.s009.docx]

| Name | |  | | Estimate | SE | t-value | DF | p-value |  | Confidence interval (95%) | |  | Simulated LRT |
| --- | --- | --- | --- | --- | --- | --- | --- | --- | --- | --- | --- | --- | --- |
|  | |  | |  |  |  |  |  |  |  |  |  |  |
|  | |  | |  |  |  |  |  |  | Lower | Upper |  | p-value |
| **Fixed effects** | |  | |  |  |  |  |  |  |  |  |  |  |
| (Intercept) | |  | | 2.620 | 4.399 | 0.595 | 527 | 0.552 |  | -6.022 | 11.262 |  |  |
| Stimulation frequency^a^ | |  | | 4.097 | 0.554 | 7.400 | 527 | <0.001 |  | 3.009 | 5.185 |  | <0.001* |
| Stimulation intensity^b^ | |  | | 0.093 | 0.041 | 2.292 | 527 | 0.022 |  | 0.013 | 0.174 |  | 0.024* |
| Location^c^ | |  | |  |  |  |  |  |  |  |  |  | 0.243 |
|  | *bladder dome* | | | -2.384 | 1.804 | -1.321 | 527 | 0.187 |  | -5.928 | 1.161 |  |  |
|  | *proximal urethra* | | | -0.692 | 1.783 | -0.388 | 527 | 0.698 |  | -4.196 | 2.811 |  |  |
|  | *membranous urethra* | | | -2.620 | 2.276 | -1.151 | 527 | 0.250 |  | -7.092 | 1.851 |  |  |
|  | *distal urethra* | | | -3.891 | 1.785 | -2.180 | 527 | 0.030 |  | -7.397 | -0.384 |  |  |
| Stimulation order^d^ | |  | |  |  |  |  |  |  |  |  |  | 0.012* |
|  | *2nd stimulation* | | | -1.442 | 0.593 | -2.433 | 527 | 0.015 |  | -2.606 | -0.278 |  |  |
|  | *3rd stimulation* | | | -1.684 | 0.605 | -2.782 | 527 | 0.006 |  | -2.873 | -0.495 |  |  |
| Age^e^ | |  | | 0.087 | 0.166 | 0.527 | 527 | 0.599 |  | -0.239 | 0.414 |  | 0.608 |
| Gender^f^ | |  | | -1.096 | 1.290 | -0.850 | 527 | 0.396 |  | -3.630 | 1.437 |  | 0.412 |
| Visit^g^ | |  | | 0.269 | 0.497 | 0.541 | 527 | 0.589 |  | -0.708 | 1.246 |  | 0.587 |
| **Random effects** | |  | |  |  |  |  |  |  |  |  |  |  |
| Group | |  | | Name | SD |  |  |  |  |  |  |  |  |
| Subject | |  | | (Intercept) | 5.135 |  |  |  |  |  |  |  |  |
| Residual | |  | |  | 5.579 |  |  |  |  |  |  |  |  |
| n | 90 | | |  |  |  |  |  |  |  |  |  |  |
| Adjusted R^2^ | 0.493 | | |  |  |  |  |  |  |  |  |  |  |
|  | | | | | | | | |  |  |  |  |  |
|  | | | | | | | | | | | | | |
|  |  |  |  |  |  |  |  |  |  |  |  |  |  |
